# Study on the mechanism of hirudin multi target delaying renal function decline in chronic kidney disease based on the “gut-kidney axis” theory

**DOI:** 10.1007/s00210-023-02888-6

**Published:** 2024-05-17

**Authors:** Chunli Long, Chenyun Zhang, Yongxiang Xie

**Affiliations:** 1 College of Basic Medicine, Guangxi University of Traditional Chinese Medicine, Nanning, China; 2 College of Graduate school, Guangxi University of Traditional Chinese Medicine, Nanning, China; 3 Department of Nephrology, The First Affiliated Hospital of Guangxi University of Traditional Chinese Medicine, No. 89-9 Dongge Rd, Qingxiu, Nanning, China

**Keywords:** Chronic kidney disease, Renal function decline, Hirudin, Gut-kidney axis, Intestinal microbiota

## Abstract

The disorder of the “gut-kidney axis” exacerbates renal function decline in chronic kidney disease (CKD), and current CKD therapy is insufficient to address this issue. Hirudin has a palliative effect on the decline of renal function. However, whether hirudin can delay CKD by regulating the “intestinal renal axis” disorder remains unclear. Unilateral ureteral ligation (UUO) induced CKD rat model, and the rats were treated with bifidobacterium and hirudin for 36 days. After 14 and 36 days of modeling, kidney and colon tissues were collected for pathology, western blot (WB) assay, and quantitative real-time PCR (qPCR) detection. Serum samples were collected for renal function testing. Fecal samples were used for 16S rRNA sequencing and research on fecal bacterial transplantation. Lipopolysaccharide combine with adenosine 5’-triphosphate (LPS + ATP)-induced intestinal epithelial cell injury was treated with a nod-like receptor pyrin domain-associated protein 3 (NLRP3) inhibitor and hirudin. Protein expression was detected using WB and qPCR. The kidneys and colons of the CKD rats exhibited varying degrees of lesions. Creatinine (CRE), blood urea nitrogen (BUN), N-acetyl-β-D-glucosidase (NAG) enzyme, and serum uremic toxins were elevated. The expression of claudin-1 and occludin was decreased, NLRP3 inflammatory-related proteins were increased, and the gut microbiota was disrupted. These pathological changes were more pronounced after 36 days of modeling. Meanwhile, high-dose hirudin treatment significantly improved these lesions and restored the intestinal flora to homeostasis in CKD rats. In vitro, hirudin demonstrated comparable effects to NLRP3 inhibitors by upregulating claudin-1 and occludin expression, and downregulating NLRP3 inflammatory-related proteins expression. The dysbiosis of the gut microbiota and impaired intestinal epithelial barrier function in CKD are associated with renal dysfunction in CKD. Hirudin delays the progression of CKD by regulating the disorder of the “gut-kidney axis” and inhibiting the activation of the NLRP3-ASC-caspase-1 pathway.

**Supplementary Information:**

The online version contains supplementary material available at 10.1007/s00210-023-02888-6.

## Introduction

Chronic kidney disease (CKD) is a serious non-communicable disease, and epidemiological surveys show that there were 697.5 million cases of CKD worldwide in 2017 (Cockwell and Fisher 2020; Collaboration 2020). The incidence of CKD in China reached 10.8% in 2012 (Zhang et al. 2012), and it has been increasing steadily over the years. Currently, CKD has become an important global public health issue, and delaying or preventing the progression of CKD is crucial in reducing the suffering of CKD patients and the economic burden on society. However, patients in the middle and late stage of CKD have prominent symptoms and multiple complications, making the current treatment methods unable to achieve satisfactory results (Wang et al. 2022b). Therefore, the development of new CKD therapies is important to alleviate patients’ symptoms and economic burden.

Meijers et al. proposed the theory of the “gut-kidney axis” in 2011 (Meijers and Evenepoel 2011), which suggests that patients with CKD experience dysbiosis in the gut microbiota, resulting in an imbalance in the gut microbiome. This imbalance exacerbates the accumulation of uremic toxins derived from the gut in the blood, leading to further deterioration of kidney function. On the other hand, dysregulated gut microbiota can disrupt the function of the intestinal epithelial barrier, enabling gut-derived uremic toxins and pathogenic bacteria to move from the gut into the bloodstream. This activates the intestinal mucosal immune system, leading to systemic inflammation and exacerbating kidney damage (Meijers and Evenepoel 2011). Clinical data shows that patients with CKD experience changes in the gut microenvironment, characterized by a decrease in anaerobic bacteria and an increase in aerobic bacteria in their feces. (Feng et al. 2021). Animal experiments have shown that treatment with probiotics increases plasma levels of short-chain fatty acids, which can help prevent kidney ischemia in mice with acute kidney injury (Feng et al. 2021). Furthermore, animals with CKD have reduced levels of tight junction (TJ) proteins in the intestinal epithelium. Adding plasma from patients with end-stage renal disease to the culture medium leads to a significant decrease in TJ proteins in cultured human intestinal cells (Vaziri et al. 2013). This suggests that kidney disease and gut microbiota and function mutually influence each other.

In addition, research has shown that inflammasomes can play a role in maintaining the balance of the gut microbiota. For example, the pro-inflammatory cytokines released by the NLRP3 inflammasome can affect the repair of intestinal epithelial injury and maintain gut homeostasis in mice (Liu et al. 2017). Meanwhile, NLRP3 inflammasomes play an important role in inducing renal inflammation and fibrosis. The mRNA levels of NLRP3 in peripheral blood mononuclear cells of dialysis-dependent CKD patients are higher than those of healthy subjects. In UUO mice with NLRP3 gene knockout, renal fibrosis, ROS damage, and apoptosis are reduced (Huang et al. 2023). The activated NLRP3-apoptosis-associated speck-like protein (ASC)-caspase-1 axis induces and secretes inflammatory cytokines, thereby contributing to the onset and progression of CKD (Franke et al. 2012). Therefore, interfering with the activation of the NLRP3 inflammasome pathway may regulate the “gut-kidney axis” and delay the progression of CKD.

Traditional Chinese Medicine (TCM) has been used in China for thousands of years and has been shown to protect people’s health. According to TCM theory, blood stasis syndrome persists throughout the course of CKD. Spleen deficiency and kidney dysfunction leading to intestinal dysbiosis and barrier damage, resulting in the production of toxic substances and blood stasis. Therefore, CKD should be treated with “promote blood circulation to dispel blood stasis” methods (Wang et al. 2022b). Hirudo medicinalis, also known as leech, is a representative Chinese medicine for promoting blood circulation to dispel blood stasis. Its main active ingredient is hirudin. Previous studies have shown that hirudin can delay the decline of renal function and improve blood stasis syndrome by inhibiting inflammation and regulating the ApoE protein in rats (Shi et al. 2019; Xie et al. 2020). However, the intervention effect of hirudin on CKD and the specific mechanism by which it intervenes in the progression of CKD are still unclear.

Therefore, this study hypothesizes that hirudin may delay the progression of CKD through regulating the “gut-renal axis” disorder and inhibiting the activation of the NLRP3 pathway, and experimental verification will be conducted. The aim is to elucidate the correlation between intestinal dysbiosis, impaired intestinal epithelial barrier function, and renal function decline in CKD, reveal the mechanism of hirudin in delaying the progression of CKD, and provide theoretical reference for the development and utilization of hirudin and the treatment of CKD.

## Materials and methods

### Animals and modeling

The specific pathogen free (SPF) male Sprague-Dawley (SD) rats (6 weeks old, 150-180 g) were purchased from the Guangxi Institute for Food and Drug Control (Nanning, Guangxi). The rats were kept in SPF animal room with alternating light and dark for 12 h/time with humidity 60% and temperature 23 ± 3 °C and free access of water. After 1 week of adaptation feeding, the rats were anesthetized with 5% pentobarbital sodium (50 mg/kg) via intraperitoneal injection. The left ureter of the left kidney was ligated to induce complete obstruction, and the muscle layer and skin were sutured and disinfected. All of the experimental procedures involving animals were approved by the Institutional Animal Care and Usage Committee (IACUC) of the Guangxi University of Chinese Medicine Institutional Review Board.

Fifteen SD rats were randomly divided into the control, molding 14-day (UUO-14d), and molding 36-day (UUO-36d) group. Another 25 SD rats were randomly divided into the control, model, high-dose hirudin (Shanghai Yuanye, China), low-dose hirudin, and bifidobacterium (State Drug License No. S20060010) group. Starting from the second day of the modeling. The rats were orally administered medication in the morning for 36 days. According to the conversion formula for the treatment dose of a 60 kg standard-weight patient to the animal standard, the low-dose hirudin was 15 mg/kg/d, the high-dose hirudin was 45 mg/kg/d, and the bifidobacterium dose was 50 mg/kg/d. After modeling again, fecal microbiota transplantation was performed on the normal model group. SD rats were divided into the normal saline enema group (NS) and the fecal microbiota transplantation group (FMT). The fecal donors for the FMT group were selected from the group receiving the most significant hirudin dose group. During the experiment, blood samples from the orbital socket and feces were collected every 2 weeks. At the end of the experiment, blood was collected from the abdominal aorta, and specimens of the colon and kidney were taken for further research.

### Cell culture and treatment

The intestinal epithelial cell line (IEC-6) was purchased from the Cell Resource Center of the Shanghai Institutes for Biological Sciences, Chinese Academy of Sciences. The cells were cultured in 90% Dulbecco’s Modified Eagle Medium (DMEM) (Thermo Fisher Scientific, USA) and 10% fetal bovine serum (FBS) (Tianhang Biotechnology, China). The culture environment is an incubator under the conditions of 5% carbon dioxide and 37 °C.

200 ng/ml lipopolysaccharide (LPS) (6 h) and 5 mmol/L adenosine 5’-triphosphate (ATP) (0.5 h) were combined to induce an in vitro model of intestinal epithelial cell injury. After modeling, hirudin was added to the cells in the model group at concentrations of 10 mg/L, 20 mg/L, 40 mg/L, 80 mg/L, 160 mg/L, and 320 mg/L, and the cells were treated for 24 h. The absorbance values were measured at 450 nm by Cell Counting Kit-8 (CCK8) (Dojindo, Japan) assay, and the high- and low-doses of hirudin were selected based on the results. The NLRP3 inhibitor CY-9 (Biolab, China) was used at a dose of 10 µm and treated for 30 min.

### Hematoxylin and eosin (H&E) staining

The colon tissue was fixed with 4% paraformaldehyde solution and then embedded with conventional dehydrated paraffin, tissue sections at a thickness of 4 μm were deparaffinized and rehydrated, stained with hematoxylin and eosin solution (ZhangYun, China), and observed for pathological changes under a light microscope.

### Transmission electron microscopy (TEM)

The colon tissue was isolated and cut into small pieces and fixed in electron microscope fixative for 4 h, washed with 0.1 M phosphate buffer (Biogot technology, China) three times, post-fixed in 1% osmium tetroxide for 2 h. It was washed with 0.1 M phosphate buffer three times, dehydrated in graded solutions of ethyl alcohol (50%, 70%, 80%, 90%, 95%, 100%), and finally embedded in epoxy resin. Ultrathin sections were cut at a thickness of 60–80 nm. Stained samples were observed under a transmission electron microscope (Nikon, Japan), and images were collected and analyzed.

### Renal function and serum uremic toxin detection

The levels of blood creatinine (CRE), blood urea nitrogen (BUN), and N-acetyl-β-D-glucosidase (NAG) in rat blood samples were detected using a fully automated biochemical analyzer (Wondfo, China). The Limulus reagent kit was used to detect the levels of LPS in rat serum. The liquid chromatography-mass spectrometry (Agilent, USA) technique was used to detect the levels of indoxyl sulfate (IS) and p-cresyl sulfate (PCS) in serum.

### Enzyme-linked immunosorbent assay (ELISA)

According to the manufacturer’s instructions, the ELISA kit (Nanjing Jiancheng, China) was uesd to test the levels of ammonia and diamine oxidase (DAO) in the serum, and measure the absorbance (OD) value at 450 nm.

### Western blot (WB) assay

Colon tissue was added to RIPA lysis buffer (Beyotime Biotechnology, batch number P0013B) and incubated at 4 °C for 30 min. Cells were added to RIPA lysis buffer on ice and lysed for 10 min. The lysate was then centrifuged at 12,000 rpm for 10 min at 4 °C, and the supernatant was collected to obtain the total protein solution. The BCA Protein Assay Kit (Beyotime Biotechnology, batch number P0010) was used to quantify the protein concentration of each sample. After quantification, protein sample buffer was added and the samples were denatured at 100 °C for 5 min. Then, equal amounts of proteins from each group were loaded onto a 10% SDS-PAGE gel and transferred to a PVDF membrane. The membranes and the primary antibody, including claudin-1 (1:1500, Abcam, ab15098), occludin (1:1000, Abcam, ab167161), ASC (1:1500, Affinity, AF6515), NLRP3 (1:1500, Affinity, DF7438), caspase-1 (1:1000, Abcam, ab179515), pro-IL-1β (1:1500, Affinity, AF4006), IL-1β (1:1500, Affinity, AF5103), IL-18 (1:1500, Abcam, ab207324), DAPDH (1:1000, Abcam, ab9485) antibody, were incubated overnight at 4 °C. The Goat anti-rabbit IgG-HRP (Jackson, batch number 111-625-144) was used for an additional 2 h at 37 °C. Finally, the protein bands were visualized using the ECL kit, and the immunoblotting signals were quantified using ImageJ software.

### Quantitative real-time PCR (qPCR)

Total ribose nucleic acid (RNA) was extracted from the colon and IEC-6 cells separately using an RNA isolation kit (BS259A, Biosharp). RNA was then reverse-transcribed into cDNA using the RevertAid RT Reverse Transcription Kit (MR101-02, Vazyme, USA). qPCR was performed at 95 °C for 120 s for initial denaturation, followed by denaturation at 95 °C for 15 s, annealing and extening at 60 °C for 30 s. This procsee was repeated for 40 cycles using the qPCR reaction kit (Q711-02, Vazyme). The gene expression levels were calculated using the 2^−△△CT^ method and normalized to the expression level of GAPDH. The sequences of the primers used in the study are disclosed in the Table 1.

**Table 1:** The sequences of the primers used in the study

Gene	Primer	Primer Sequences
GAPDH	Forward	TGTGTCCTGGCACACGTTTC
	Reverse	GATGGTGATGGGTTTCCCGT
NLRP3	Forward	ACGGCAAGTTCGAAAAAGGC
	Reverse	CTTGCTGACTGAGGACCTGA
ASC	Forward	AGACATGGGCATACAGGAGC
	Reverse	GCAATGAGTGCTTGCCTGTG
caspase−1	Forward	AAGAAGGTGGCGCATTTCCT
	Reverse	GACGTGTACGAGTGGGTGTT
IL−1β	Forward	AGGATTGCTTCCAAGCCCTTGACT
	Reverse	ACAGCTTCTCCACAGCCACCATGA
IL−18	Forward	ATGCCTGATATCGACCGAAC
	Reverse	TGTGTCCTGGCACACGTTTC

### 16S rRNA sequencing

The DNA of gut microbiota was extracted by using the E. Z. N. A. soil DNA Kit (Omega Bio-Tek, Norcross, GA, U.S.). The primers used for bacterial 16S rRNA gene amplification were as follows: the forward primer pair was 338 F: 5’-ACTCCTACGGGAGGCAGCAG-3’ and the reverse primer was 806 R: 5’-GGACTACHVGGGTWTCTAAT-3’. The instrument was used to amplify the hypervariable region V3-V4 of bacterial 16S rRNA gene is an ABI GeneAmp 9700 PCR thermocycler (ABI, CA, USA). The amplification and sequencing steps were roughly divided into three steps. First of all, the PCR amplification of the 16S rRNA gene was performed as follows: initial denaturation at 95 °C for 3 min, followed by 27 cycles of denaturing at 95 °C for 30 s, annealing at 55 °C for 30 s, and extension at 72 °C for 45 s, and single extension at 72 °C for 10 min, and end at 4 °C. Subsequently, the PCR product was extracted from 2% agarose gel and purified by using the AxyPrep DNA Gel Extraction Kit (Axygen Biosciences, USA) according to the manufacturer’s instructions and quantified using Quantus Fluorometer (Promega, USA). Finally, purified amplicons were pooled in equimolar and paired-end sequenced on an Illumina MiSeq PE300 platform/NovaSeq PE250 platform (Illumina, San Diego, USA) according to the standard protocols by Majorbio Bio-Pharm Technology Co. Ltd. (Shanghai, China).

The raw 16S rRNA gene sequencing reads obtained were demultiplexed and quality-filtered by FASTP (version 0.20.0). Subsequently, the raw 16S rRNA gene sequencing reads were merged by FLASH (version 1.2.7) with the following three conditions: (1) the 300 bp reads were truncated at any site receiving an average quality score of < 20 over a 50 bp sliding window, and the truncated reads shorter than 50 bp were discarded, reads containing ambiguous characters were also discarded; (2) only overlapping sequences longer than 10 bp were assembled according to their overlapped sequence. The maximum mismatch ratio of the overlap region is 0.2. Reads that could not be assembled were discarded; and (3) Samples were distinguished according to the barcode and primers, and the sequence direction was adjusted for exact barcode matching, and 2 nucleotide mismatches in primer matching. Operational taxonomic units (OTUs) with a 97% similarity cutoff were clustered using UPARSE, and chimeric sequences were identified and removed. The taxonomy of each OTU representative sequence was analyzed by RDP Classifier against the 16S rRNA database using a confidence threshold of 0.7.

### Statistical analysis

SPSS 20.0 software was used for data analysis and all data were expressed as mean ± standard deviation (SD). The comparison between two groups of quantitative data is conducted using t-test, and one-way analysis of variance was used for comparison of multiple groups of measurement indicators. *P* < 0.05 meant that the difference was statistically significant.

## Results

### UUO causes renal lesions and hypoplasia in rats

**Fig. 1:**
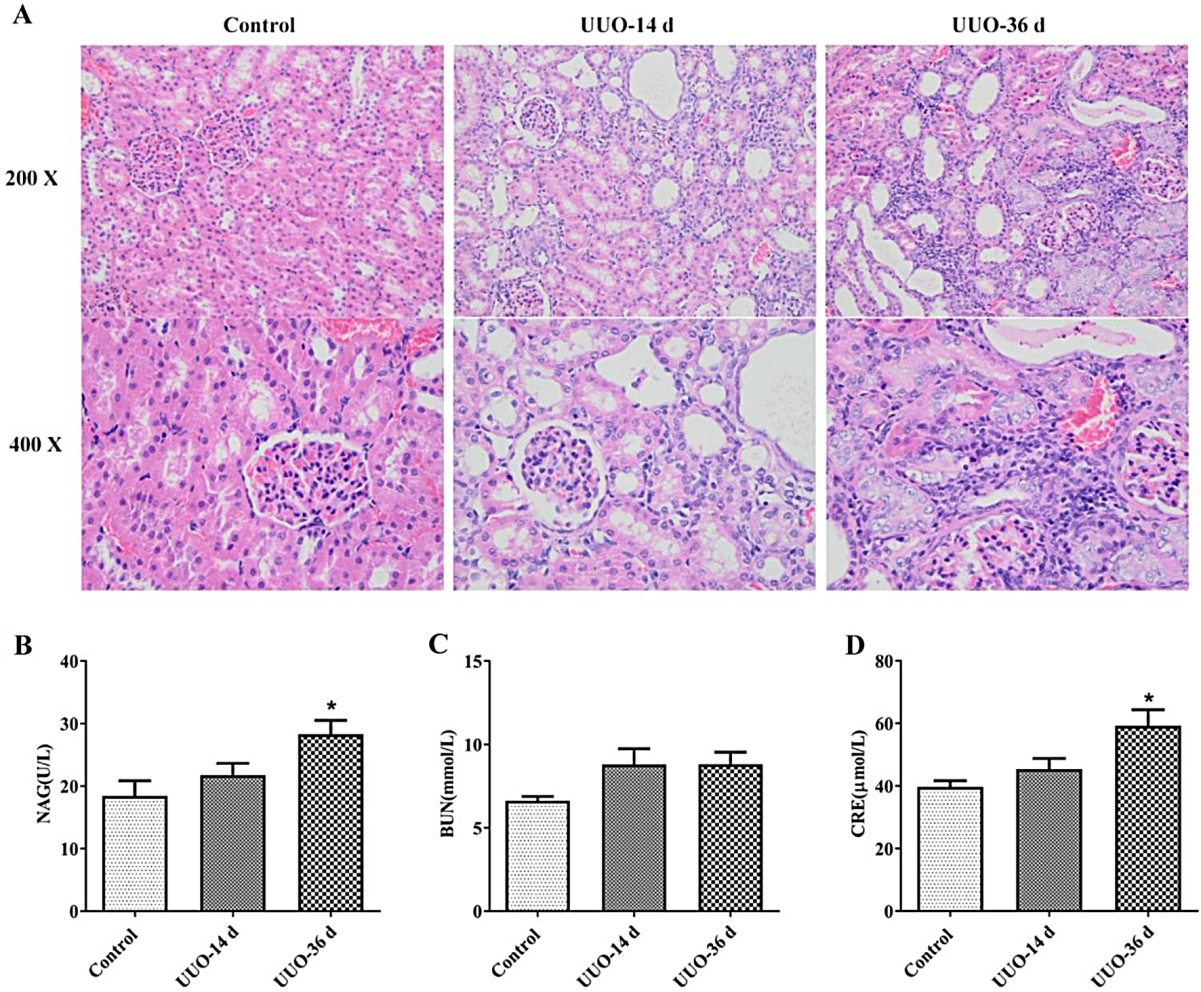
UUO causes renal lesions and hypoplasia in rats. HE staining of rat kidney (**A**). Changes in levels of renal function indicators NAG (**B**), BUN (**C**), and CRE (**D**) in rat blood samples. ^*^P < 0.05

Compared with the control group, rat kidneys showed a diffuse proliferation of the glomerular stroma, swelling of tubular epithelial cells, tubular atrophy, and a small amount of inflammatory cell infiltration after 14 days of modeling. Renal injury was further exacerbated after 36 days of modeling, with severe swelling of renal tubular epithelial cells and a large number of infiltrating inflammatory cells (Fig. 1A). With the prolongation of the modeling time, the levels of NAG, BUN, and CRE gradually increased in the blood of rats (Fig. 1B–D).

### UUO disrupts intestinal mucosal barrier and intestinal flora homeostasis, and activates NLRP3 inflammasome pathway in rats

**Fig. 2:**
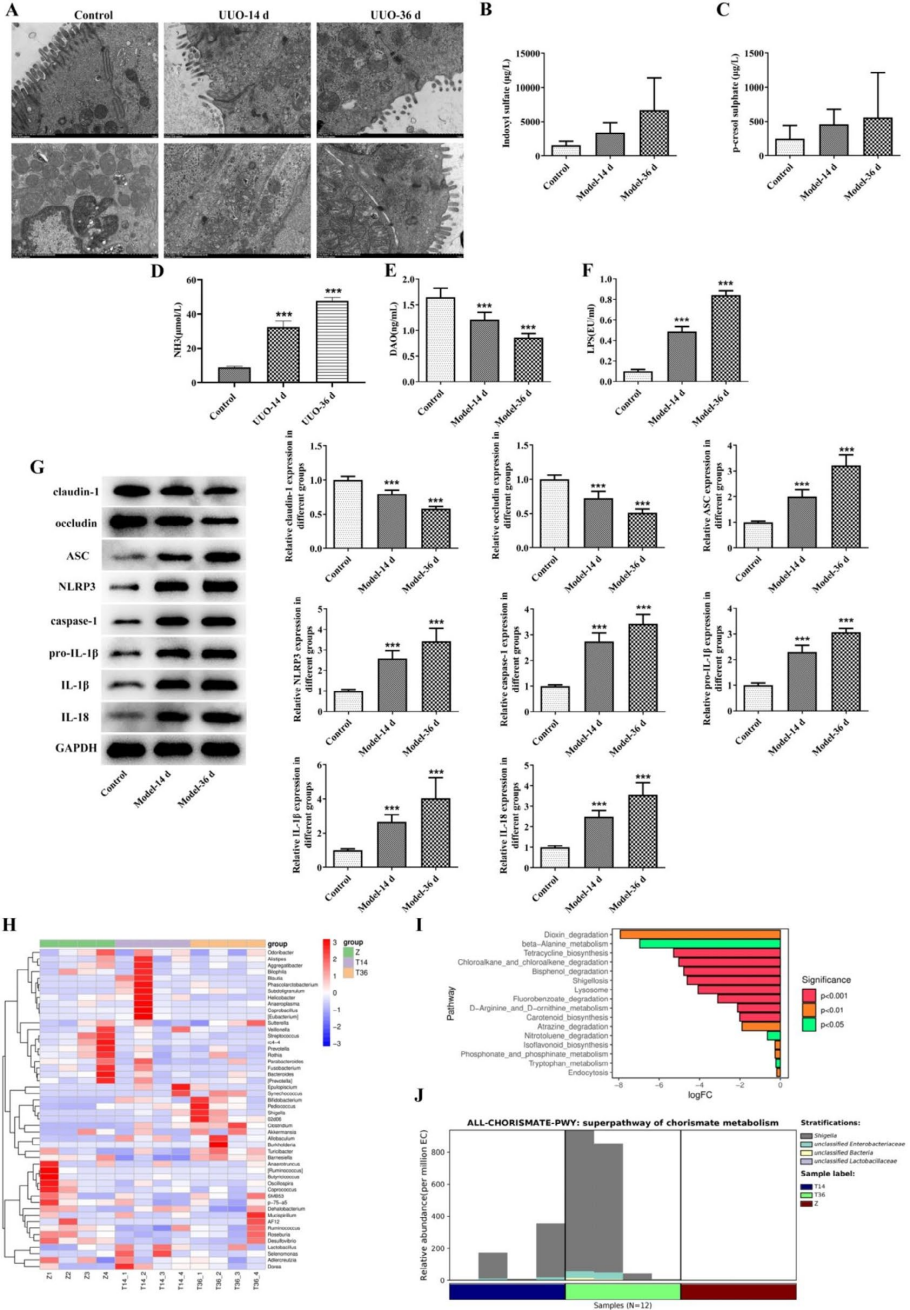
UUO disrupts intestinal mucosal barrier and intestinal flora homeostasis, and activates NLRP3 inflammasome pathway in rats. Colonic transmission electron microscopy observation (**A**). Changes in serum IS (**B**), PCS (**C**), NH3 (**D**), DAO (**E**), and LPS (**F**) levels.WB detection of claudin-1, occludin, ASC, NLRP3, Caspase-1, Pro-IL-1β, IL-1β, and IL-18 protein expression in the colon tissue (**G**). Heatmap of genus-level species composition for species clustering of the intestinal flora (**H**), graph of taxonomic units displaying differences between groups (**I**), and graph of KEGG metabolic pathways for differences between groups in the modeling 36-day group and the control group (**J**). ^***^P < 0.00

As shown in Fig. 2A, the colons of rats in the UUO-14d group began to exhibit lesions such as villous atrophy and mitochondrial vacuolation. After 36 days, the colon mucosal damage worsened, with sparse villi, increased mitochondrial vacuolation, and disappeared and fracture of the ridges. Compared to the control group, as the modeling time increased, the levels of serum IS, PCS, LPS, and blood NH3 in rats gradually increased, while the content of DAO decreased (Fig. 2B–F), indicating that UUO caused intestinal barrier damage and dysbiosis in rats.

Next, we examined the expression of tight junction and NLRP3 inflammasome-related proteins in the rat colon. The results showed that with the prolongation of modeling time, the expression of claudin-1 and occludin gradually decreased, while the expression of ASC, NLRP3, Caspase-1, IL-1β, and IL-18 gradually increased (Fig. 2G). 16S RNA sequencing showed that UUO induced intestinal bacterial flora disorders in rats, with fecal *Bacteroides*, *Bifidobacterium*, *Ruminococcus*, *Clostridium*, *Lactobacillus*, and other flora genus level species abundances in the feces being significantly altered compared with the control group (Fig. 2H-I). Metabolic pathways such as Tetracycline biosynthesis, Chloroalkane and chloroalkene degradation, Bisphenol degradation, and Isoflavonoid biosynthesis were significantly downregulated after 36 days of modeling (Fig. 2J).

### Hirudin ameliorates renal and colonic lesions and inhibits NLRP3 inflammasome pathway activation in CKD rats

**Fig. 3:**
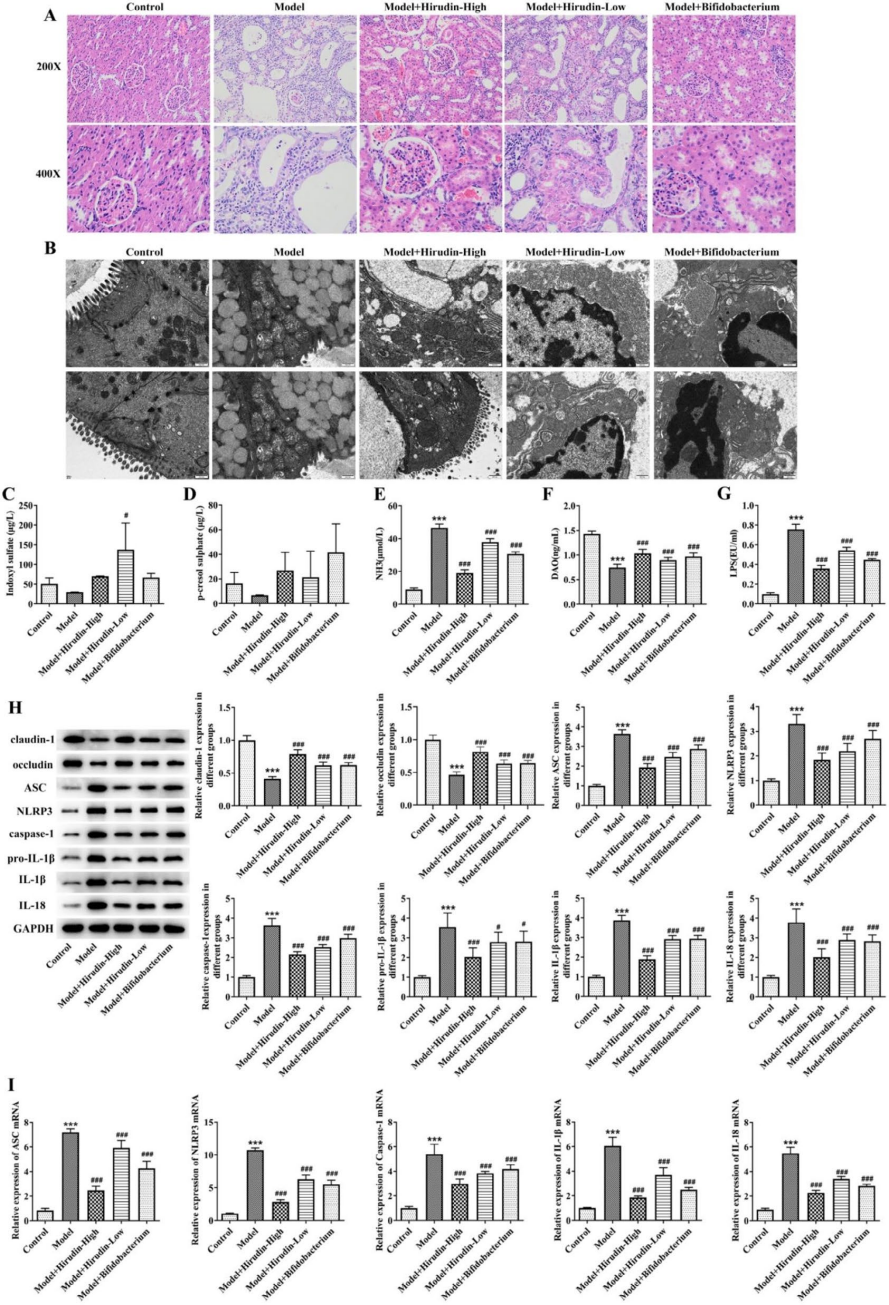
Hirudin ameliorates renal and colonic lesions and inhibits NLRP3 inflammasome pathway activation in CKD rats. HE staining of kidney (**A**) and colon transmission electron microscopy (**B**) in each group of rats. Changes in serum IS (**C**), PCS (**D**), NH3 (**E**), DAO (**F**) and LPS (**G**) levels in rats of each group. WB detection of claudin-1, occludin, ASC, NLRP3, Caspase-1, Pro-IL-1β, IL-1β, and IL-18 protein expression in colon of each group (**H**). qPCR detection of ASC, NLRP3, Caspase-1, IL-1β and IL-18 mRNA levels in colon of each group (**I**). ^*^P < 0.05, ^**^P < 0.01, ^***^P < 0.001 vs. Control; ^#^*P* < 0.05, ^##^*P* < 0.01, ^###^*P* < 0.01 vs. Model

Based on the above results, the CKD rat model after UUO-36d was selected for further study. As shown in Fig. 3A,B, the low-dose of hirudin slightly ameliorated kidney and colon damage in CKD rats, while the high-dose of hirudin and Bifidobacterium significantly improved renal and colonic lesions in CKD rats, as evidenced by a significant reduction in renal tubular epithelial cell swelling and inflammatory cell infiltration, and an essentially intact mitochondrial crest in the colon with no significant vacuolation and a neatly organized villus. Compared with the model group, hirudin treatment dose-dependently downregulated the levels of IS, PCS, NH3, and LPS, and increased the content of DAO in CKD rats (Fig. 3C–G). In addition, hirudin treatment dose-dependently increased the expression of claudin-1 and occludin, and decreased the expression of ASC, NLRP3, Caspase-1, IL-1β, and IL-18 in the colon of CKD rats (Fig. 3H). And qPCR detection showed that with the increase of hirudin dose, the mRNA expression of ASC, NLRP3, Caspase-1, IL-1β, and IL-18 significantly decreased (Fig. 3I).

### Hirudin promotes intestinal flora balance in CKD rats

**Fig. 4:**
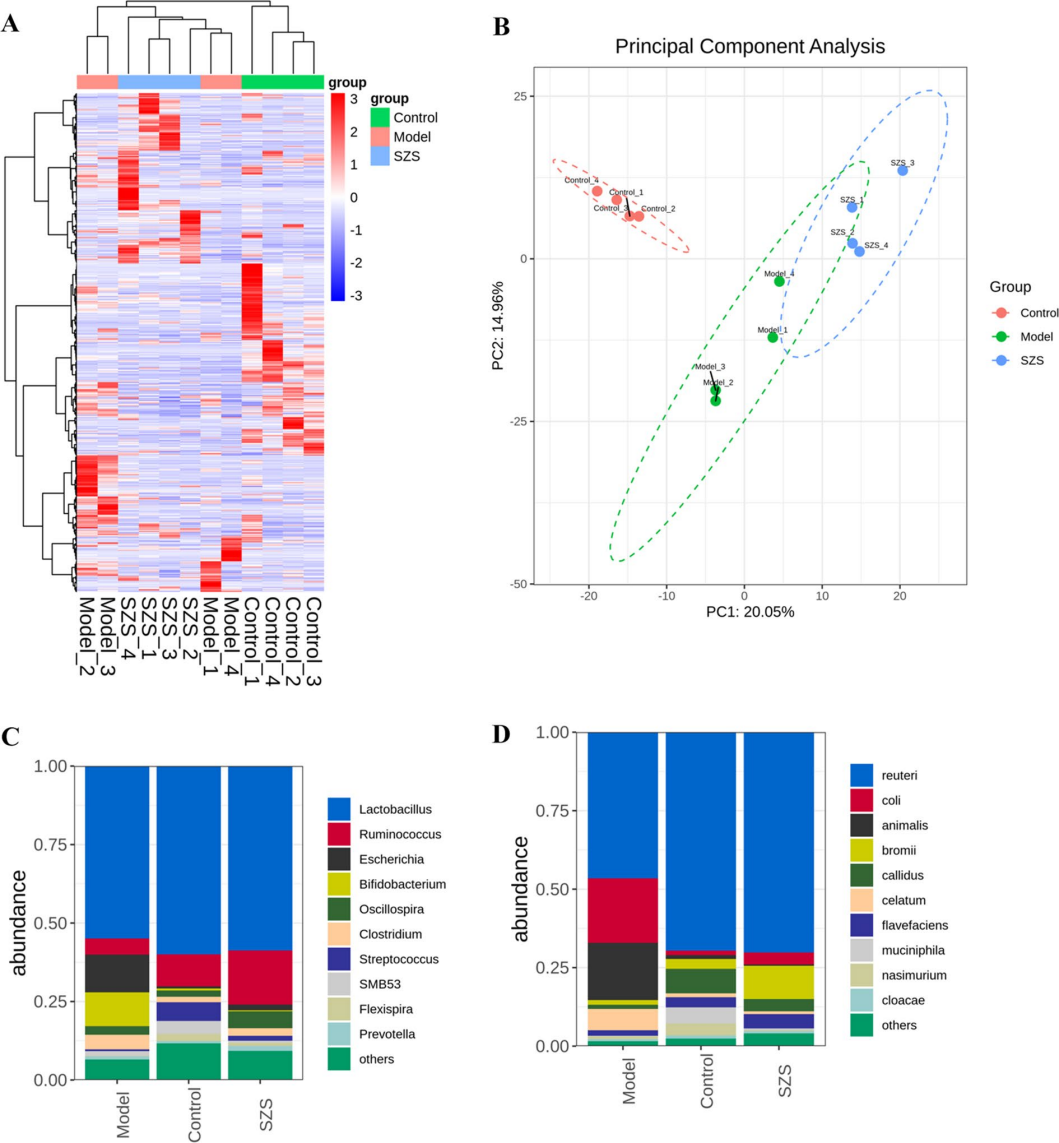
Hirudin promotes intestinal flora balance in CKD rats. Heatmap of the abundance of OTUs in the gut flora (**A**) and PCA analysis of OTUs abundance (**B**). Histograms of the mean relative abundance of flora species at the genus (**C**) and species (**D**) level

Combining the above results, the effect of high-dose hirudin was the most significant. Therefore, the feces of rats in the high-dose hirudin group, the control, and model groups were selected and tested for 16S intestinal flora. The results showed that the total species abundance of the intestinal flora OUTs differed between the control and model groups of rats (Fig. 4A), and PCA analysis at the OTUs level showed that there was a clear separation of the community composition of the control and model groups of flora, and that high-dose hirudin treatment regulated the composition of the intestinal flora in CKD rats (Fig. 4B). At the genus level, the relative abundance of *Lactobacillus*, *Ruminalococcu*s, and *Streptococcus* decreased and the relative abundance of *Escherichia*, *Bifidobacterium*, and *Clostridium* increased in the model group, and hirudin was able to regulate the relative abundance of *Lactobacillus*, *Escherichia, Streptococcus*, and *Clostridium* to normalize (Fig. 4C). At the species level, hirudin was able to regulate the relative abundance of *Lactobacillus reuteri*, *Escherichia col*i, *Ruminococcus callidus*, and *Clostridium celatum* to normalize (Fig. 4D).

### Hirudin improves epithelial barrier function and inhibits NLRP3 inflammasome pathway activation by modulating intestinal flora

**Fig. 5:**
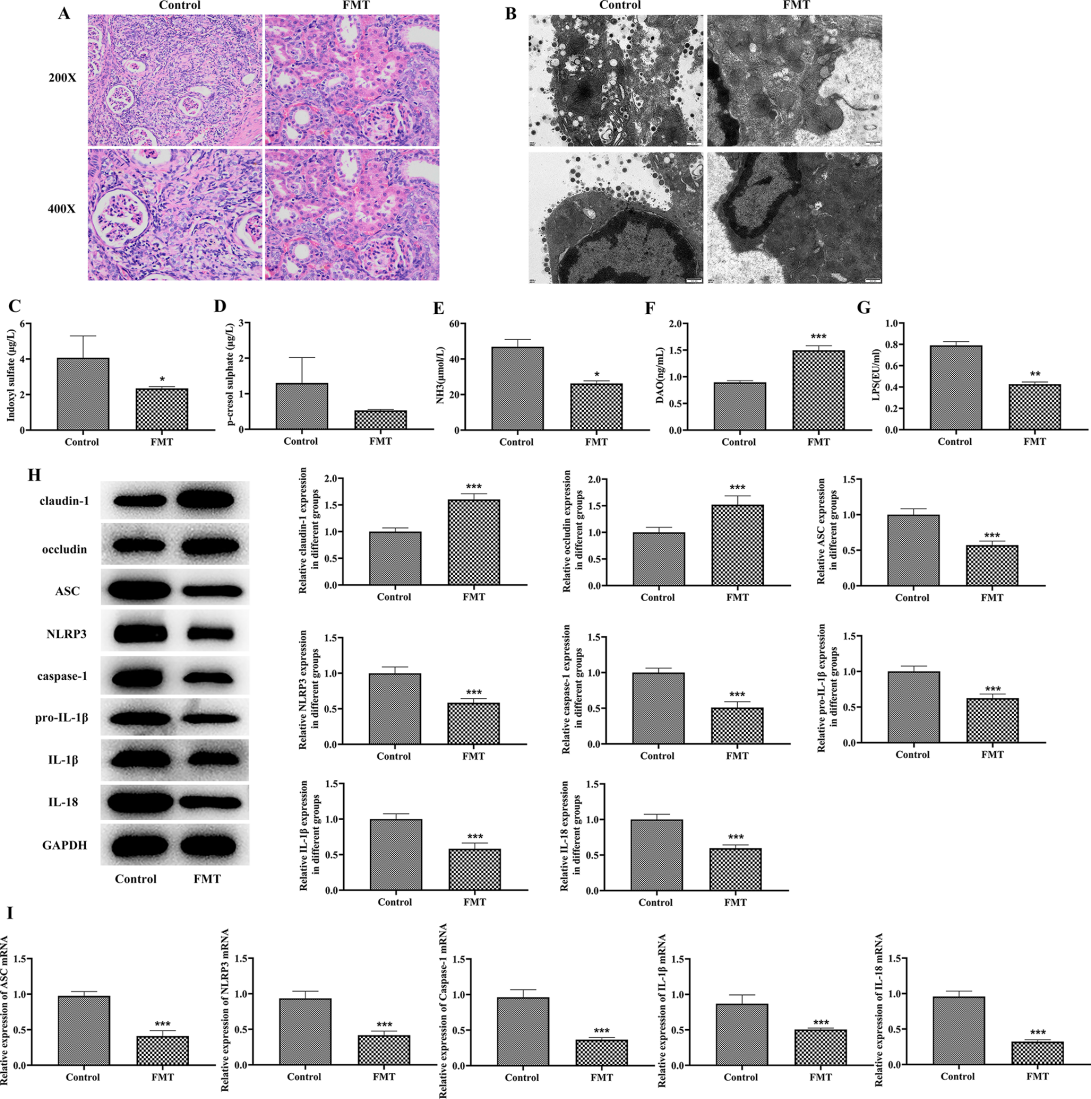
Hirudin improves epithelial barrier function and inhibits NLRP3 inflammasome pathway activation by modulating intestinal flora. HE staining of kidney (**A**) and colonic electron microscopy (**B**) observations in saline and fecal transplantation (FMT) groups of rats. Changes in serum IS (**C**), PCS (**D**), NH3 (**E**), DAO (**F**) and LPS (**G**) levels in rats. WB detection of claudin-1, occludin, ASC, NLRP3, Caspase-1, Pro-IL-1β, IL-1β, and IL-18 protein expression in colon of each group (**H**). qPCR detection of ASC, NLRP3, Caspase-1, IL-1β and IL-18 mRNA levels in colon of each group (**I**). ^*^P < 0.05, ^**^P < 0.01, ^***^P < 0.001

In experiments involving fecal microbiota transplantation (FMT), rats treated with normal saline (NS) exhibited severe kidney and colon lesions, tubular atrophy, extensive infiltration of inflammatory cells, and severe colon mitochondrial vacuolization. Conversely, rats in the FMT group showed reduced kidney and colon lesions (Fig. 5A,B). Furthermore, compared to the NS group, rats in the FMT group demonstrated decreased levels of IS, PCS, LPS, and NH3, as well as increased levels of DAO (Fig. 5C–G). WB and qPCR analysis revealed that the expression of claudin-1 and occludin proteins were significantly increased, and ASC, NLRP3, caspase-1, IL-1β, IL-18 protein and mRNA expression were significantly decreased in the FMT group (Fig. 5H,I).

### Hirudin repairs intestinal epithelial barrier and delays CKD progression by inhibiting the NLRP3 inflammasome pathway

**Fig. 6:**
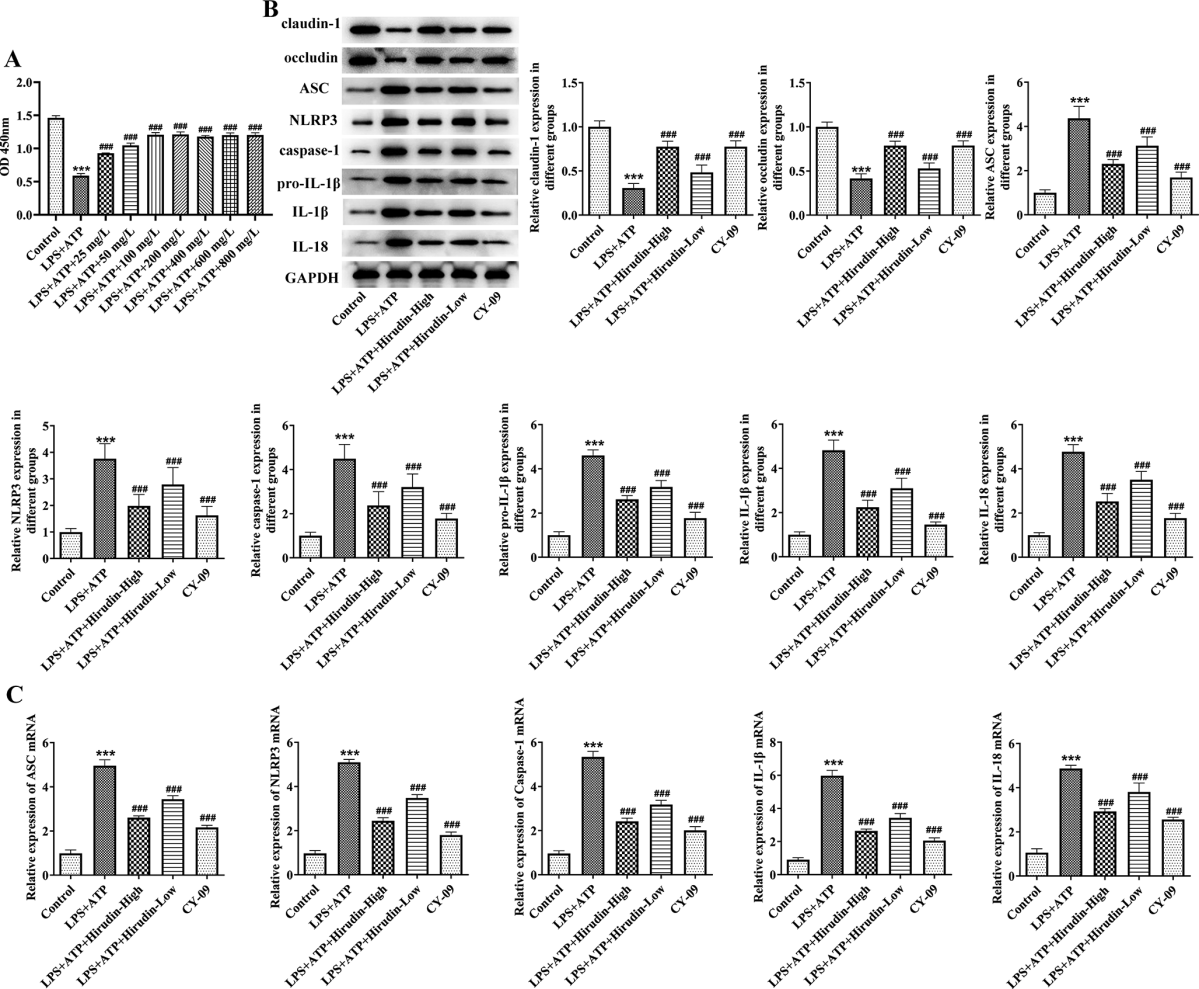
Hirudin repairs intestinal epithelial barrier by inhibiting the NLRP3 inflammasome pathway. CCK8 assay to screen for appropriate hirudin treatment concentration (**A**). WB assay for claudin-1, occludin, ASC, NLRP3, Caspase-1, Pro-IL-1β, IL-1β, and IL-18 protein expression in IEC-6 cells (**B**). qPCR assay for ASC, NLRP3 in IEC-6 cells, Caspase-1, IL-1β and IL-18 mRNA levels in IEC-6 cells (**C**). ^***^P < 0.001 vs. Control; ^###^*P* < 0.001 vs. Model

As shown in Fig. 6A, the viability of IEC-6 cells decreased after LPS + ATP induction. After treatment with different concentrations of hirudin, cell viability gradually increased (Fig. 6A). Based on the results, concentrations of 25 mg/L and 100 mg/L of hirudin were selected as low and high doses for subsequent experiments. The results showed that LPS + ATP induction significantly decreased the expression of claudin-1 and occludin, and increased the expression of ASC, NLRP3, Caspase-1, IL-1β, and IL-18 proteins and mRNA in IEC-6 cells. With the dose of hirudin increased, claudin-1 and occludin proteins expression were notably increased, and ASC, NLRP3, Caspase-1, IL-1β, IL-18 protein and mRNA expression were significantly decreased, which presenting the same effect as that of the NLRP3 inhibitor CY-9 (Fig. 6B,C).

## Discussion

CKD is a common disease that seriously threatens human health, and the current treatments are not sufficient for all patients (Lambers Heerspink and de Zeeuw 2013; Noone and Licht 2014; Wang et al. 2022b). This study is based on the theory of the “gut-kidney axis” and investigates the correlation between gut microbiota imbalance, impaired intestinal epithelial barrier function, activation of the inflammasome pathway, and the progression of CKD renal function through in vivo and in vitro experiments. It also elucidates the intervention effect of hirudin on the decline of CKD renal function.

Studies have shown that the colon is one of the major organs for the production of uremic toxins, and enterogenic uremic toxins are closely associated with progression and death in CKD (Aronov et al. 2011). It has been found that CKD patients are prone to disorders of gastrointestinal function and intestinal microecology (Sabatino et al. 2015). The increase in pathogenic bacteria in the intestines caused by the imbalance of intestinal microbiota is directly related to the production of intestinal-derived uremic toxins (Armani et al. 2017; Meijers and Evenepoel 2011). Intestinal-derived uremic toxins IS and PCS induce oxidative stress and inflammatory reactions, contributing to the continuous deterioration of kidney disease (Fukagawa and Watanabe 2011). Also, imbalance of intestinal microbiota can induce damage to the intestinal epithelial barrier, leading to the translocation of LPS into the bloodstream and exacerbating renal immunoinflammatory damage (Armani et al. 2017). The present study showed that rats with CKD had elevated levels of CRE, BUN, NAG, IS, PCS, and LPS, decreased expression of colonic tight junction proteins claudin-1 and occludin, and significantly altered abundance of fecal species at the genus level of *Bacteroides*, *Bifidobacterium*, and *Lactobacillus* compared to the control group. These results indicate that there is an imbalance in the intestinal microbiota and impairment of the intestinal epithelial barrier function in CKD rats, which is closely related to the decline in renal function in CKD. Furthermore, high-dose hirudin treatment significantly ameliorated kidney and colon damage, reversed the expression of related factors and proteins, and regulated the homeostasis of intestinal flora in CKD rats. This suggests that hirudin can regulate the imbalance of the “gut-kidney axis” in CKD, repair the intestinal epithelial barrier function, and delay the decline in renal function in CKD.

In addition, studies have shown that the inflammasome may play a role in maintaining microecological homeostasis by regulating intestinal flora. Among them, the NLRP3 inflammasome is the most representative, and the cytokines released by the NLRP3 inflammasome, such as IL-1β and IL-18, play an important role in repairing intestinal epithelial damage and maintaining intestinal homeostasis (Liu et al. 2017; Sutterwala et al. 2014). Meanwhile, the NLRP3 inflammasome induced renal inflammatory responses both glomerular cells, tubular cells, renal interstitium, and infiltrating inflammatory cells. NLRP3 was involved in the onset and progression of renal diseases (Vilaysane et al. 2010; Wang et al. 2022a). Suggests that interfering with the activation of the NLRP3 inflammasome pathway is important for repairing the intestinal barrier and delaying the progression of CKD. Our results showed a significant increase in the levels of ASC, NLRP3, caspase-1, IL-1β, and IL-18 proteins and mRNA in the colon of CKD rats and the LPS + ATP-induced injury model of colonic epithelial cells, suggesting that the activation of the NLRP3-ASC-caspase-1 inflammasome pathway is associated with impaired intestinal epithelial barrier function and renal tubular damage. However, with the increase in hirudin dosage, the protein and mRNA levels of ASC, NLRP3, Caspase-1, IL-1β, and IL-18 were significantly reduced both in vivo and in vitro. This indicates that hirudin can inhibit the activation of the NLRP3-ASC-caspase-1 inflammasome pathway, thereby delaying the decline in renal function in CKD.

In summary, this study revealed the relationship between gut microbiota imbalance, impaired intestinal epithelial barrier function, activation of the NLRP3 inflammasome pathway, and the decline in renal function in CKD. It elucidated that hirudin restores gut microbiota homeostasis, repairs the intestinal epithelial barrier, and delays the decline in renal function in CKD by regulating the “gut-kidney axis” disorder and inhibiting the activation of the NLRP3-ASC-caspase-1 inflammasome pathway. This provides a new insight into the treatment of CKD with traditional Chinese medicine.

## Electronic supplementary material

Below is the link to the electronic supplementary material.

Supplementary Material 1

Supplementary Material 2

Supplementary Material 3

## Data Availability

The datasets generated for this study are available on request to the corresponding author.
