# Supplementary material for: Study on the mechanism of hirudin multi target delaying renal function decline in chronic kidney disease based on the “gut-kidney axis” theory
Source: Naunyn Schmiedebergs Arch Pharmacol. 2024 May 17;397(10):7951–62. doi: 10.1007/s00210-023-02888-6 (PMC11450085; doi:10.1007/s00210-023-02888-6)
Supplement: Supplementary file 1 — Supplementary Material 1 [file 210_2023_2888_MOESM1_ESM.docx]

**Full uncropped Gels and Blots image(s)**

**Figure 2**

claudin-1


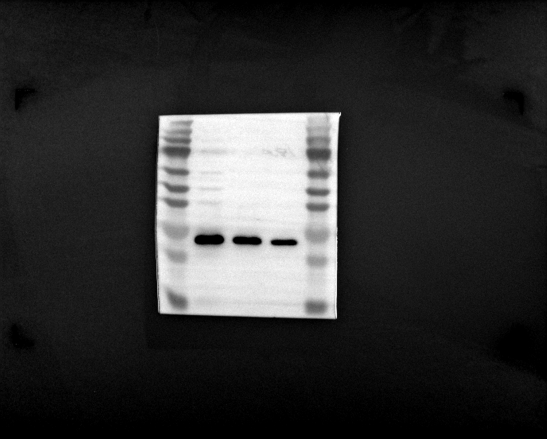

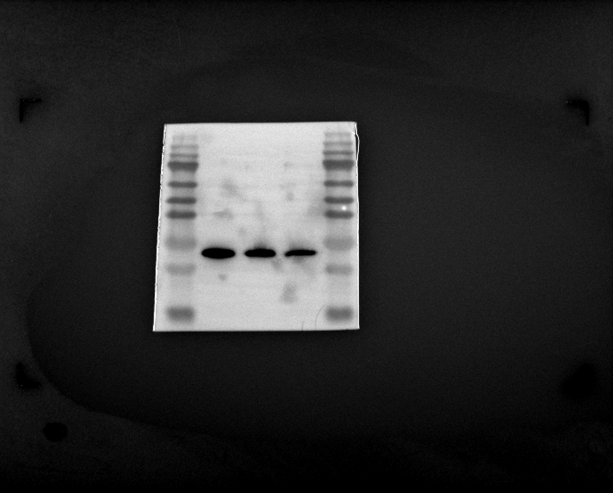

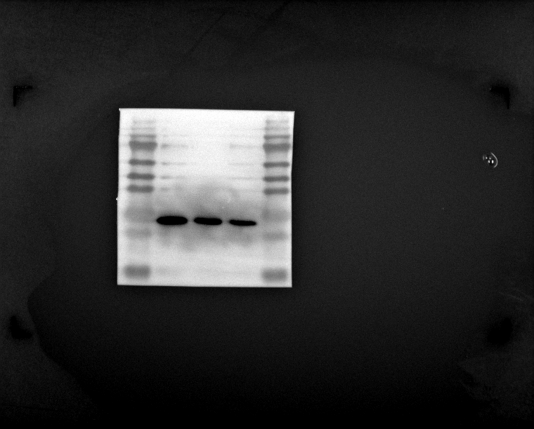


Occludin


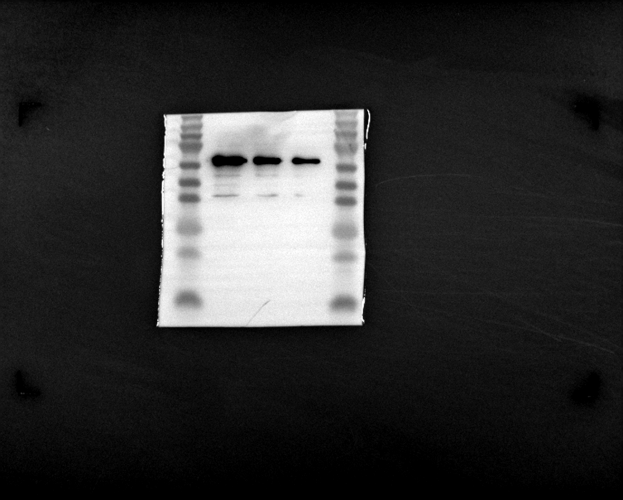

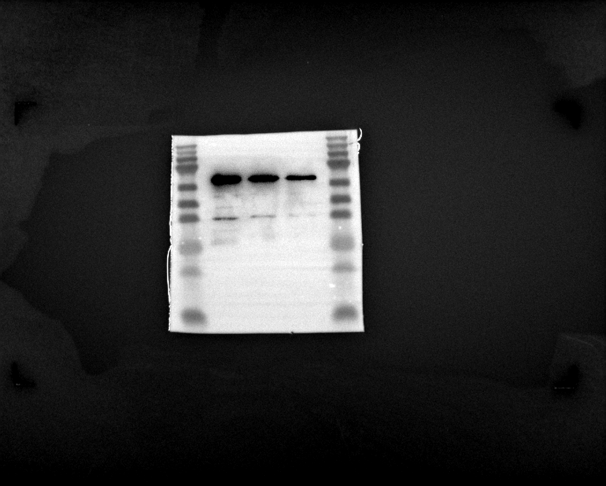

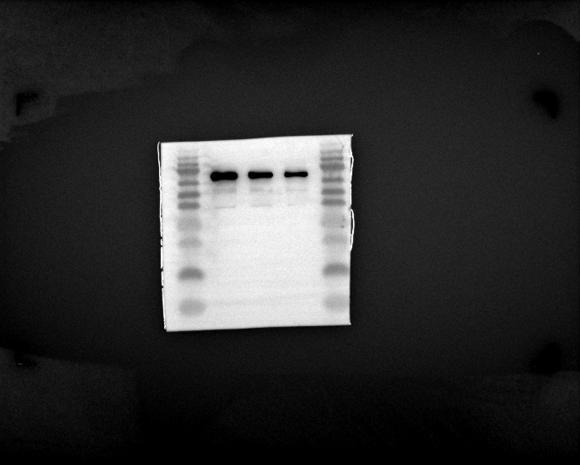


ASC:


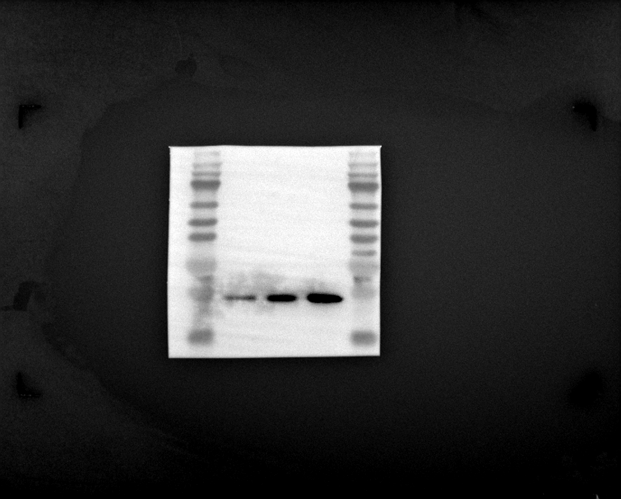

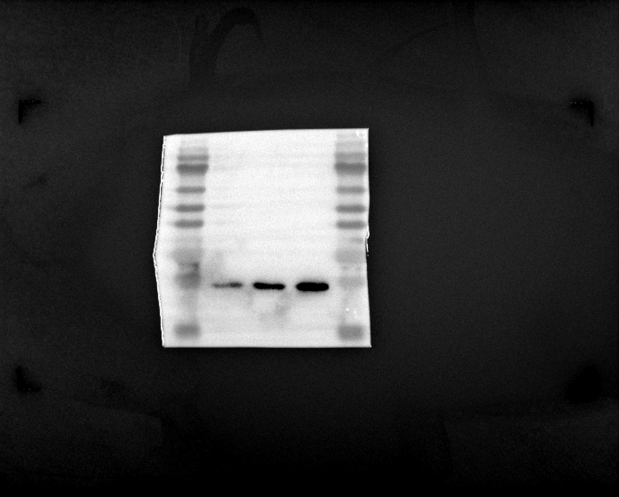

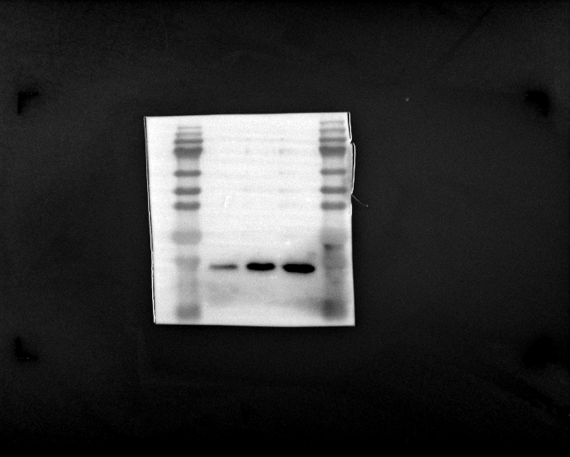


Caspase-1


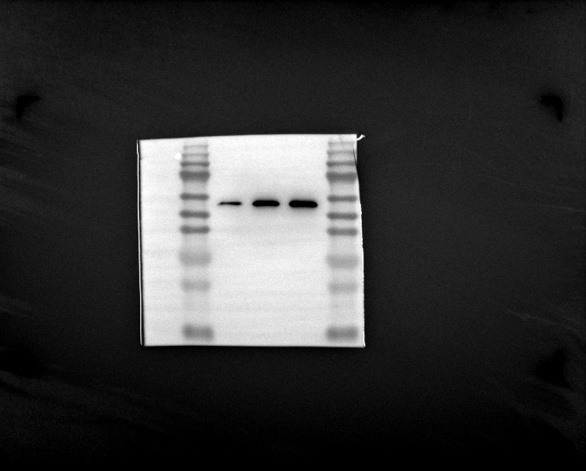

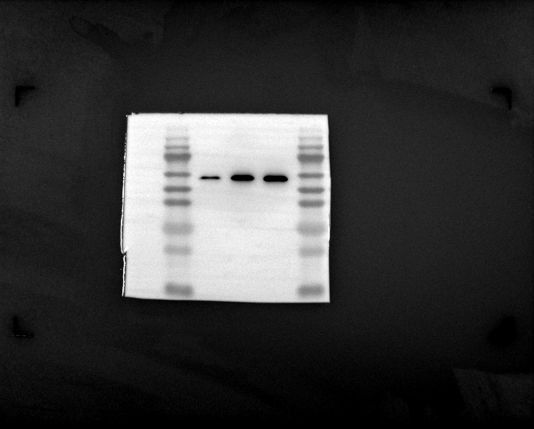

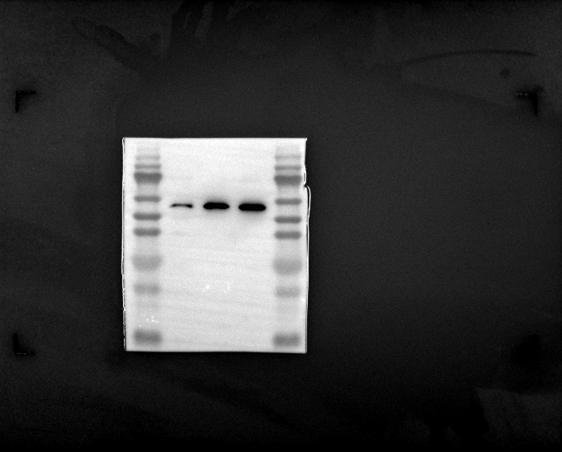


NLRP3


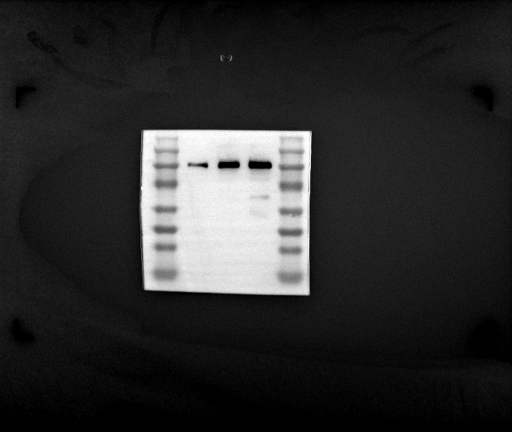

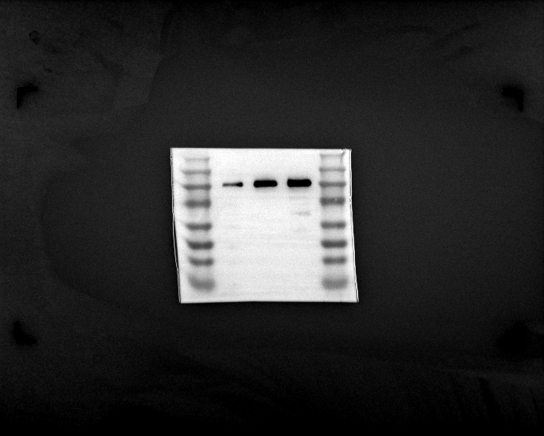

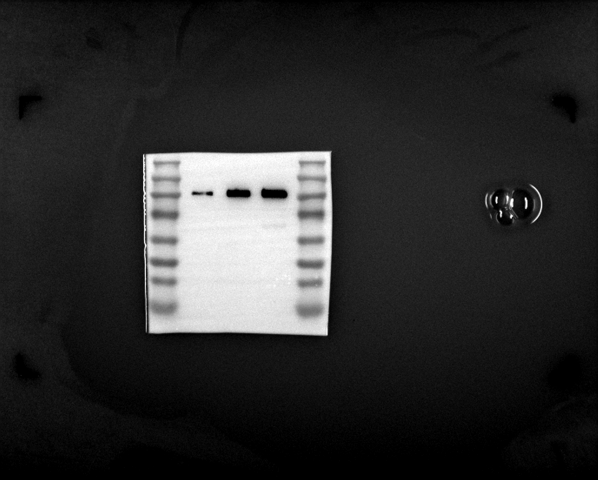


Pro-IL-1β


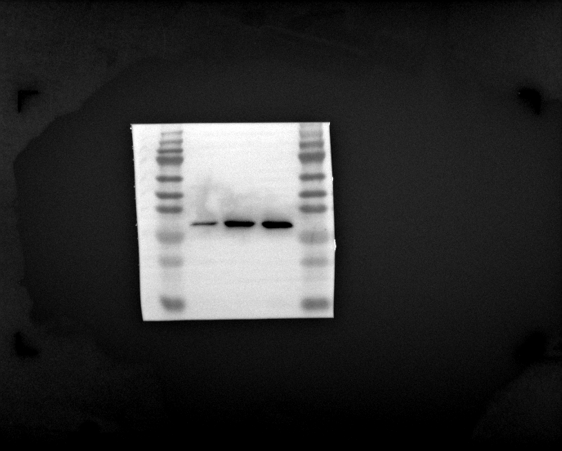

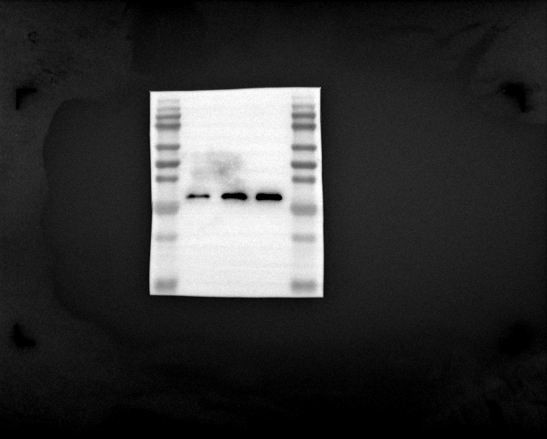

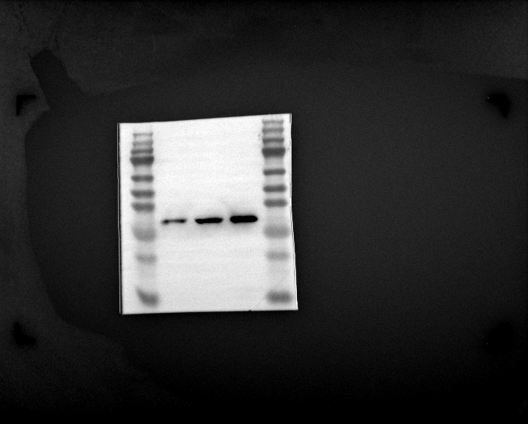


IL-1β


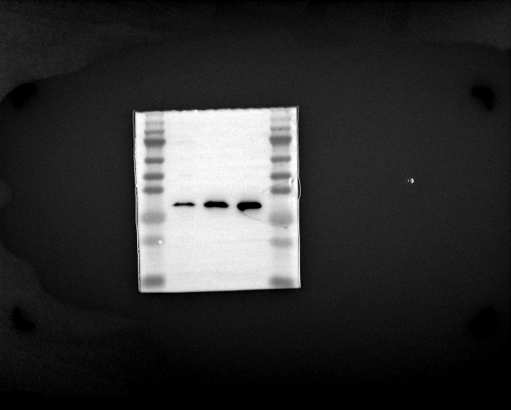

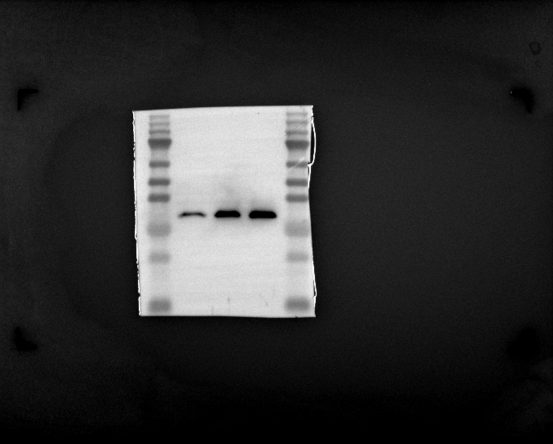

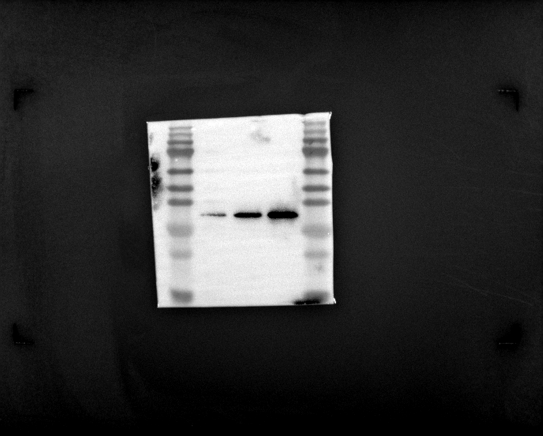


IL-18


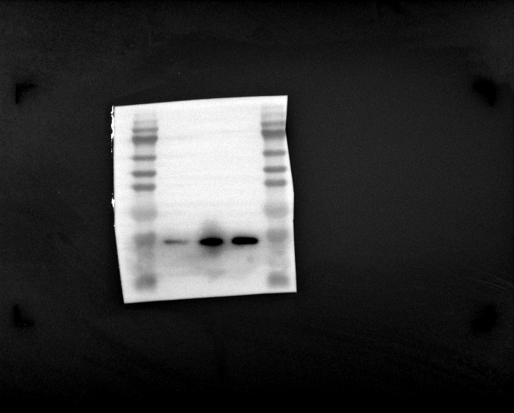

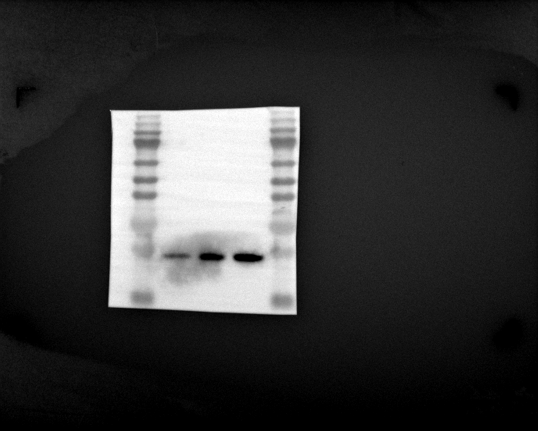

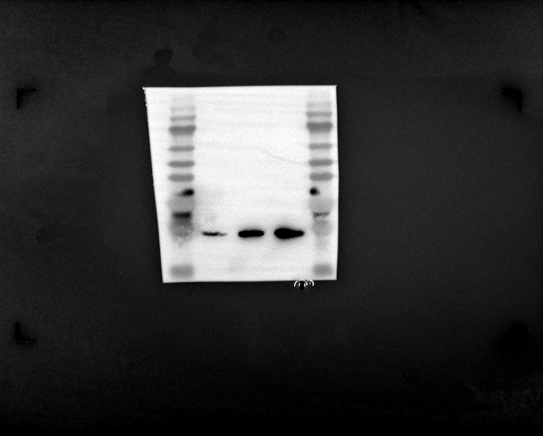


GAPDH


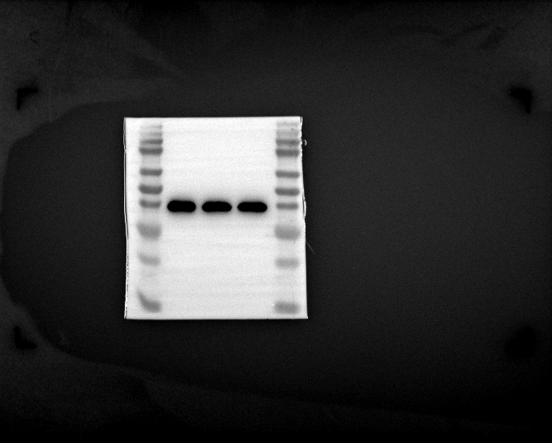

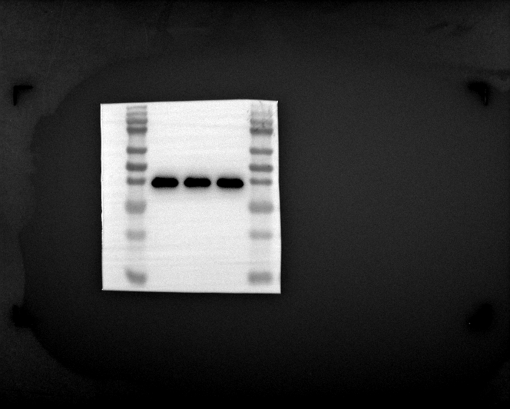

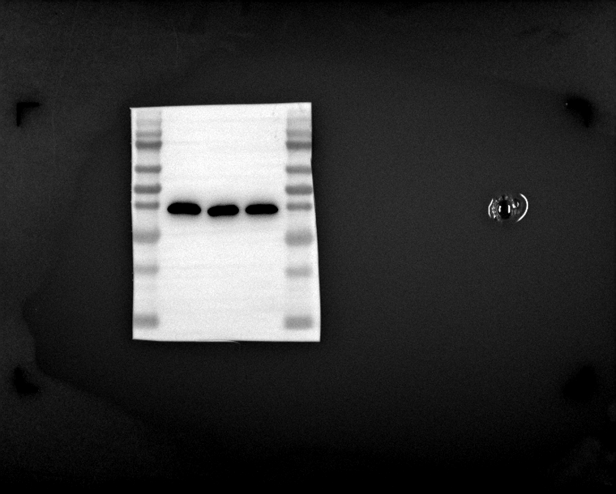


**Figure 3**

claudin-1


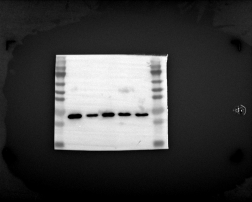

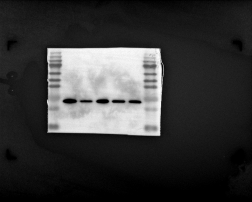

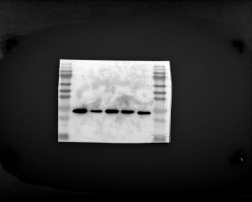


Occludin


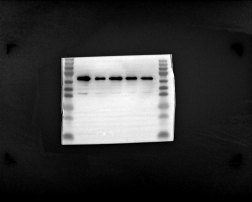

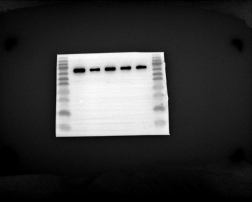

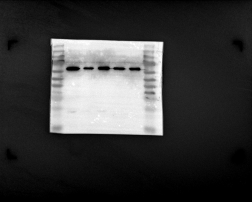


ASC


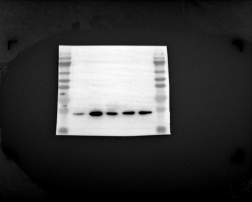

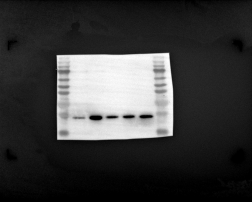

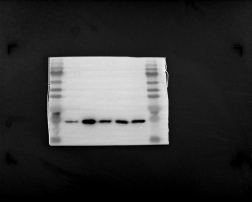


NLRP3


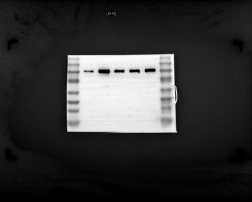

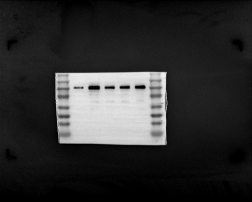

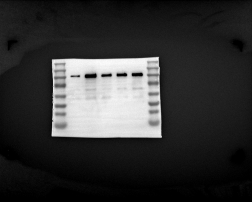


Caspase-1


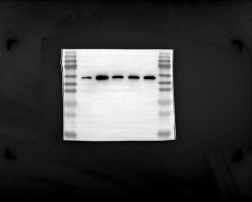

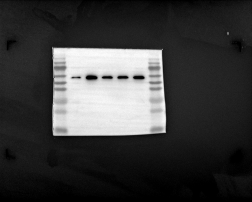

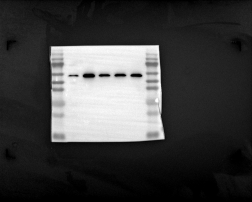


Pro-IL-1β


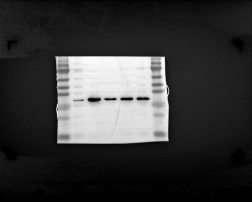

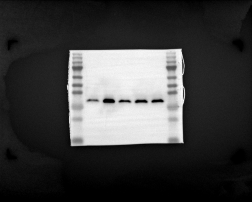

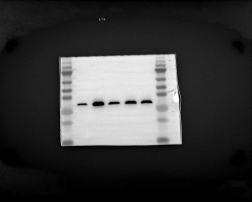


IL-1β


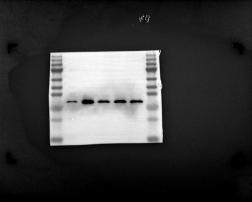

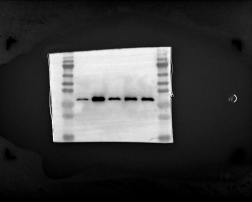

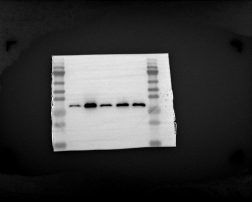


IL-18


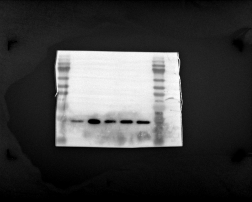

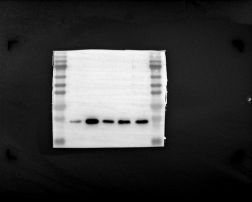

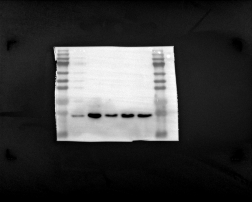


GAPDH


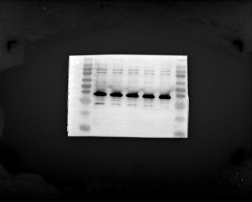

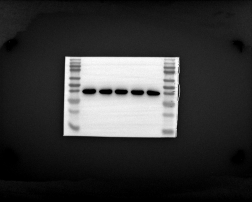

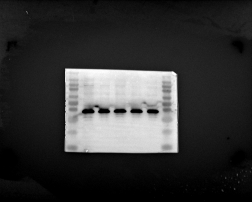


**Figure 5**

claudin-1


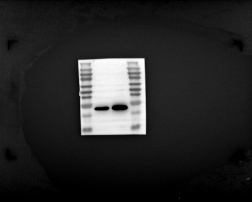

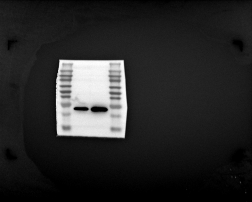

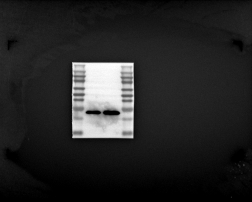


Occludin


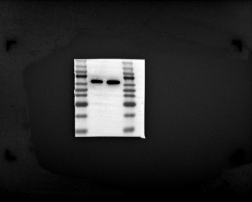

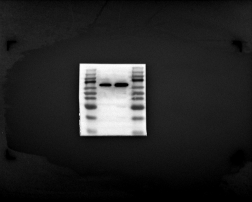

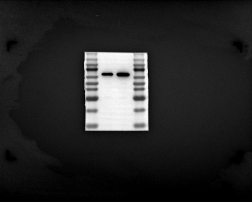


ASC


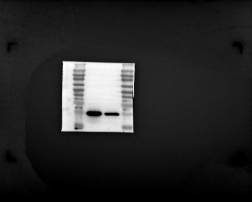

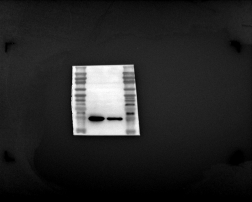

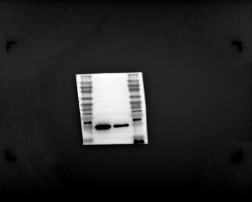


NLRP3


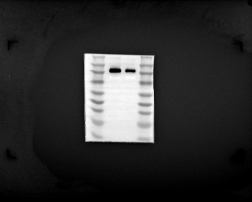

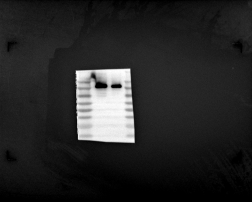

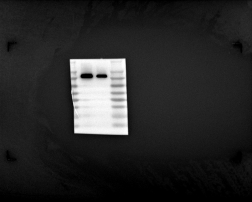


Caspase-1


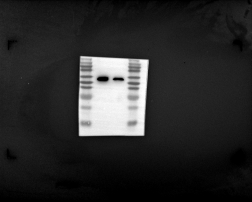

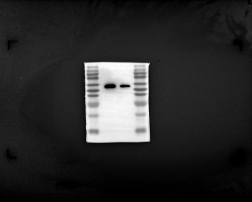

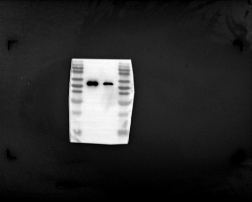


Pro-IL-1β


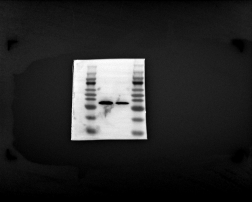

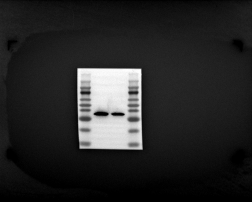

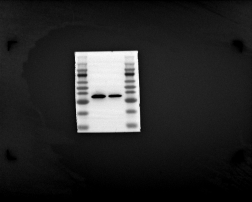


IL-1β


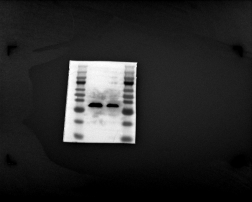

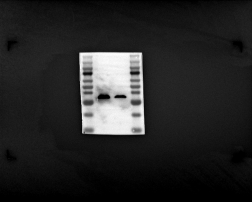

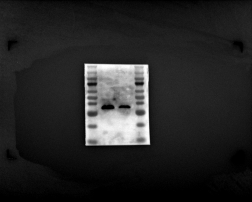


IL-18

**
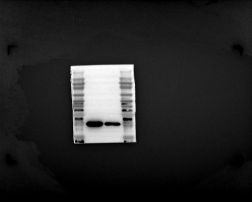

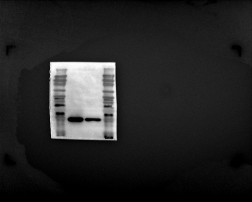

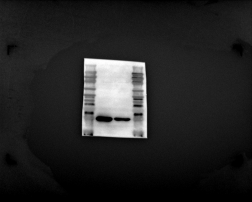
**

GAPDH

**
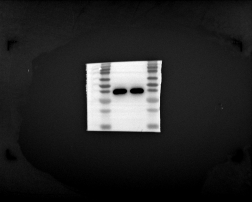

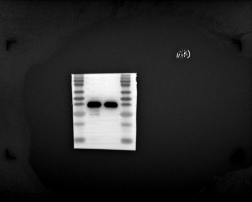

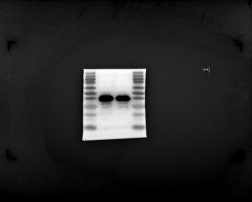
**

**Figure 6**

claudin-1


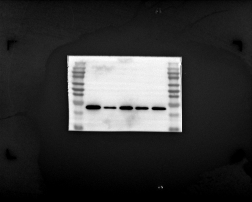

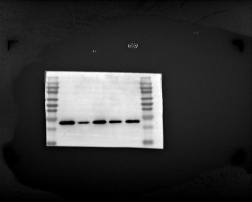

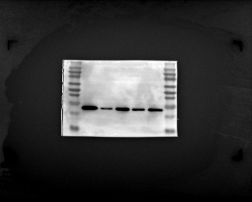


Occludin


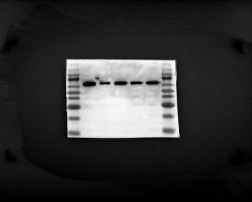

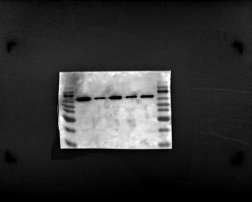

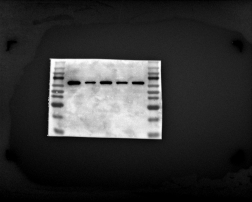


ASC


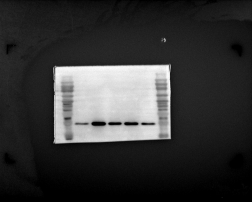

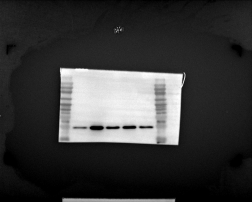

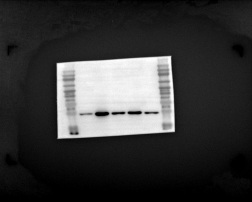


NLRP3


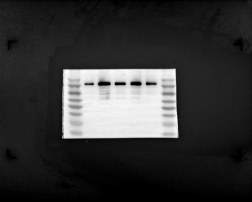

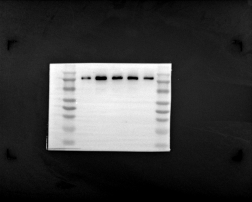

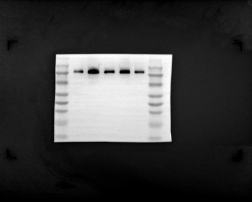


Caspase-1


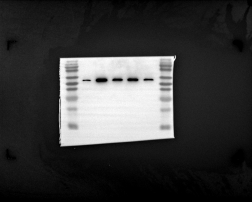

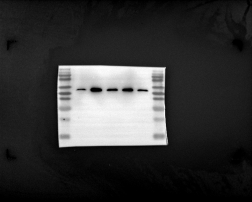

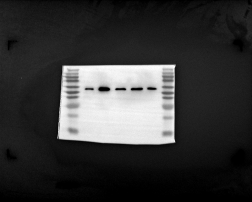


Pro-IL-1β


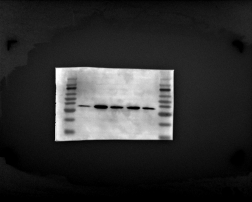

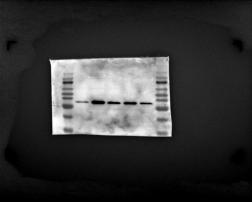

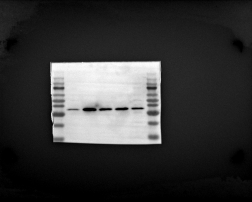


IL-1β


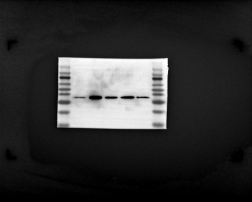


IL-18

GAPDH
