# Supplementary material for: Study on the mechanism of hirudin multi target delaying renal function decline in chronic kidney disease based on the “gut-kidney axis” theory
Source: Naunyn Schmiedebergs Arch Pharmacol. 2024 May 17;397(10):7951–62. doi: 10.1007/s00210-023-02888-6 (PMC11450085; doi:10.1007/s00210-023-02888-6)

Original TEM image

**Figure 2A**

Control


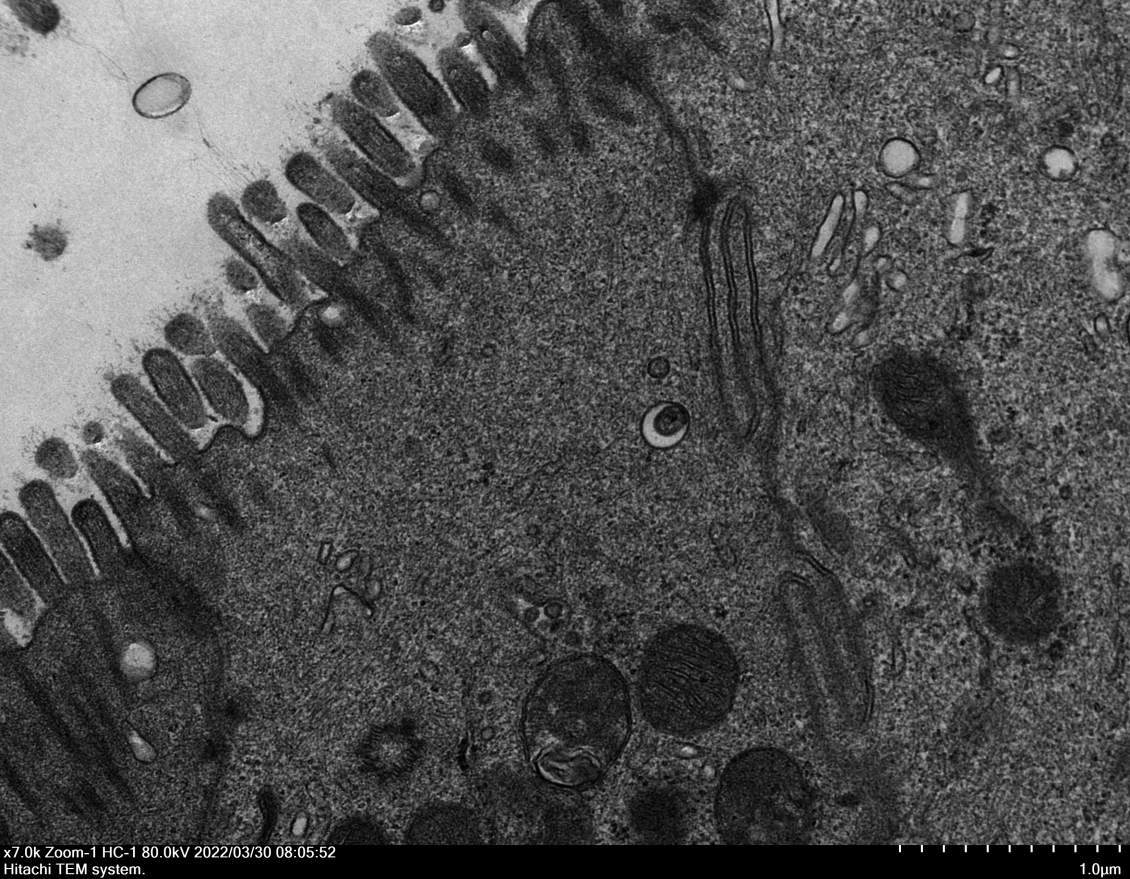


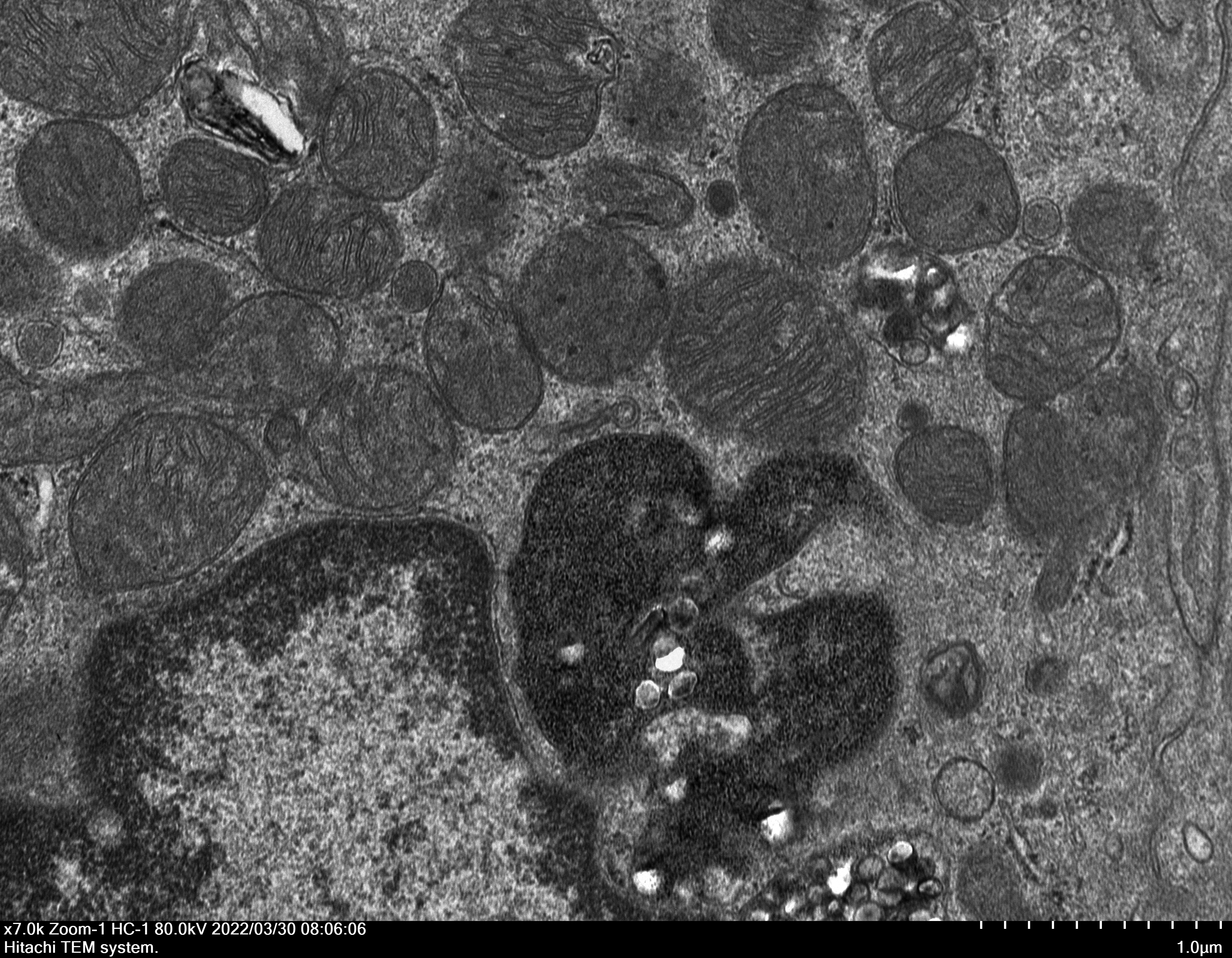


UUO-14d


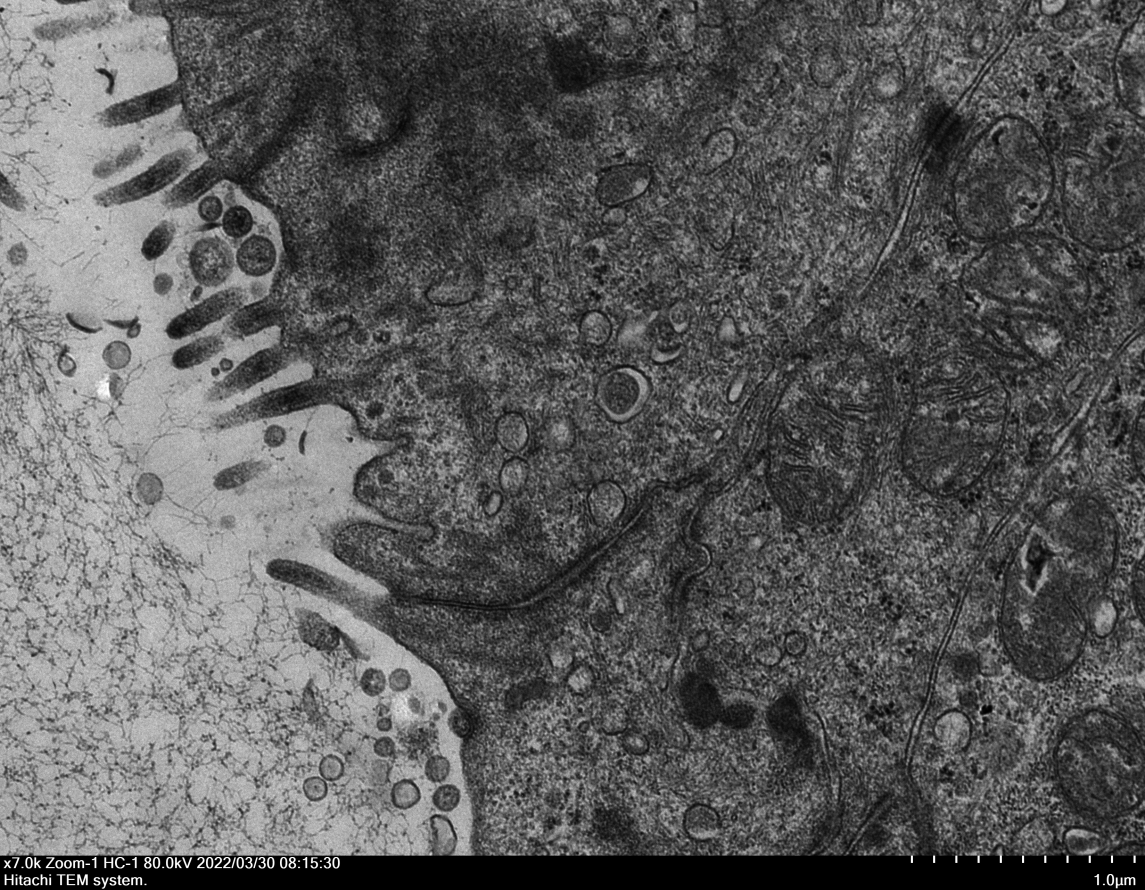


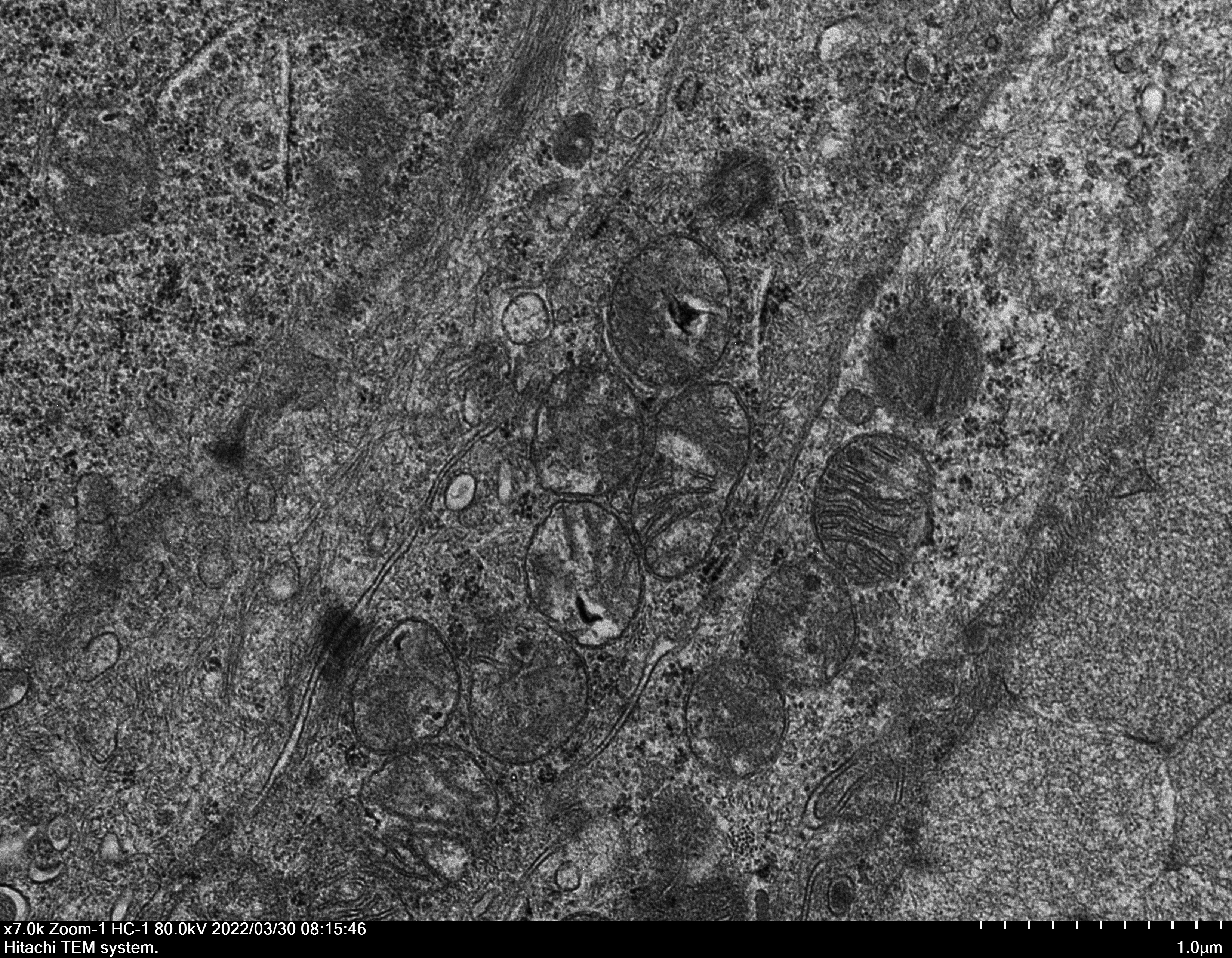


UUO-36d

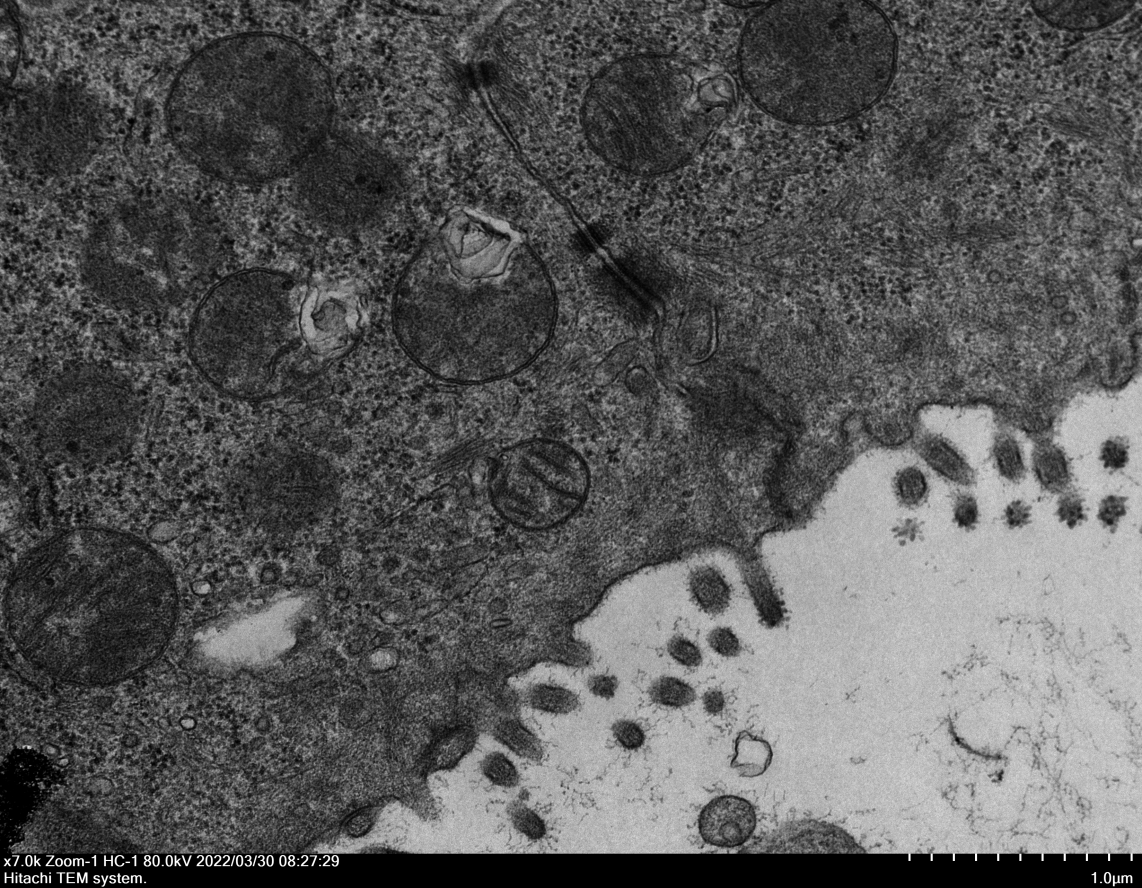


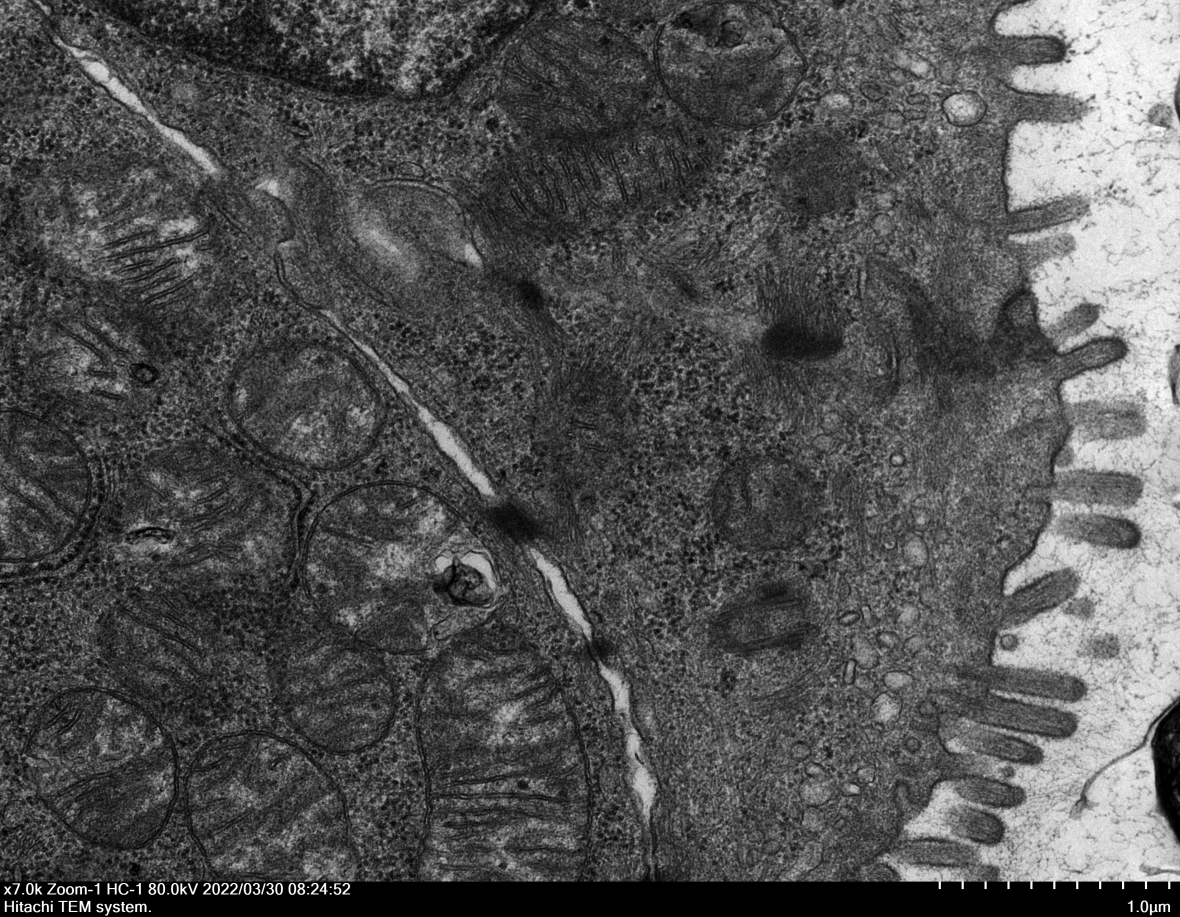


**Figure 3B**

Control


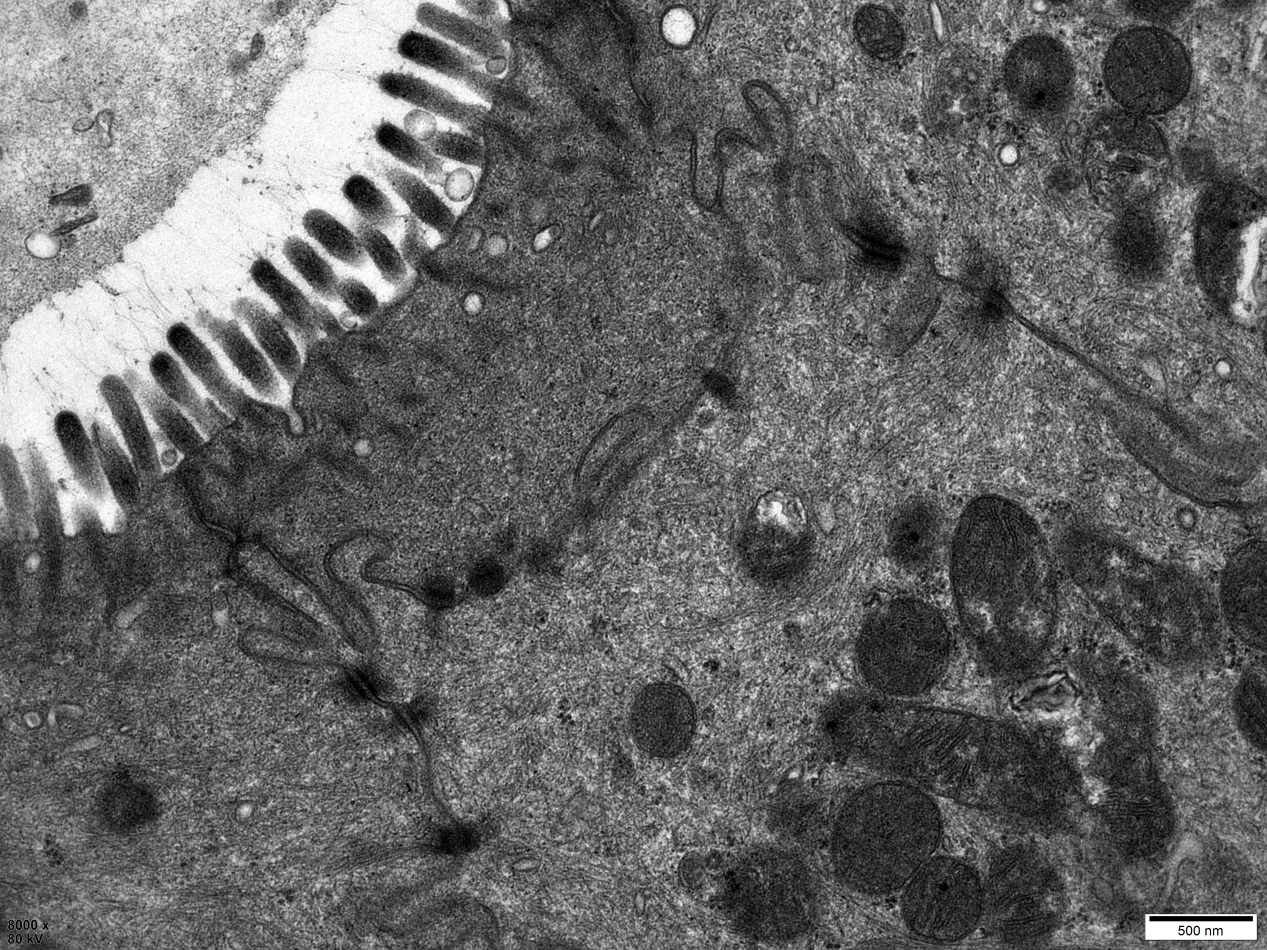


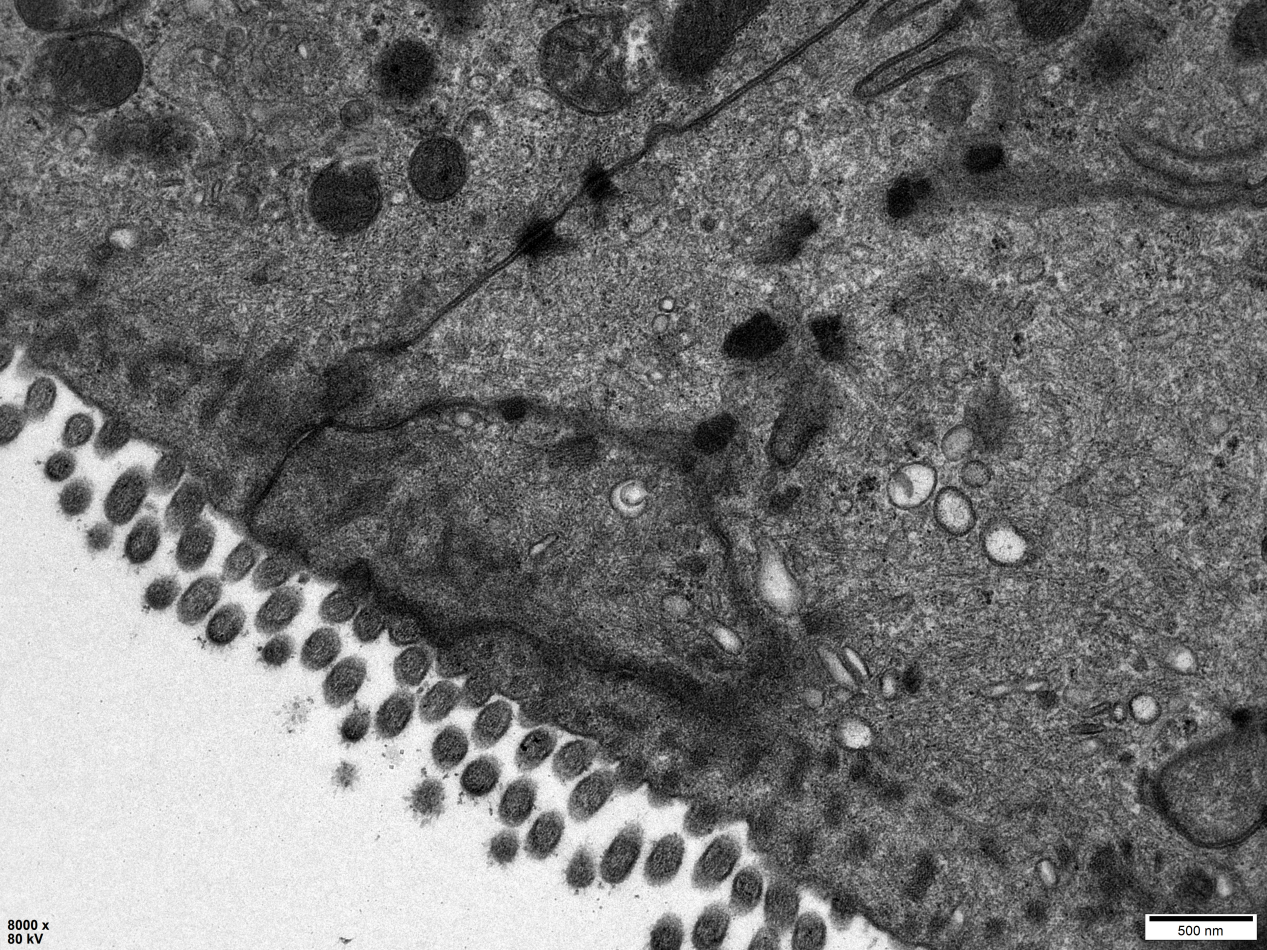


Model

Model+Hirudin-High


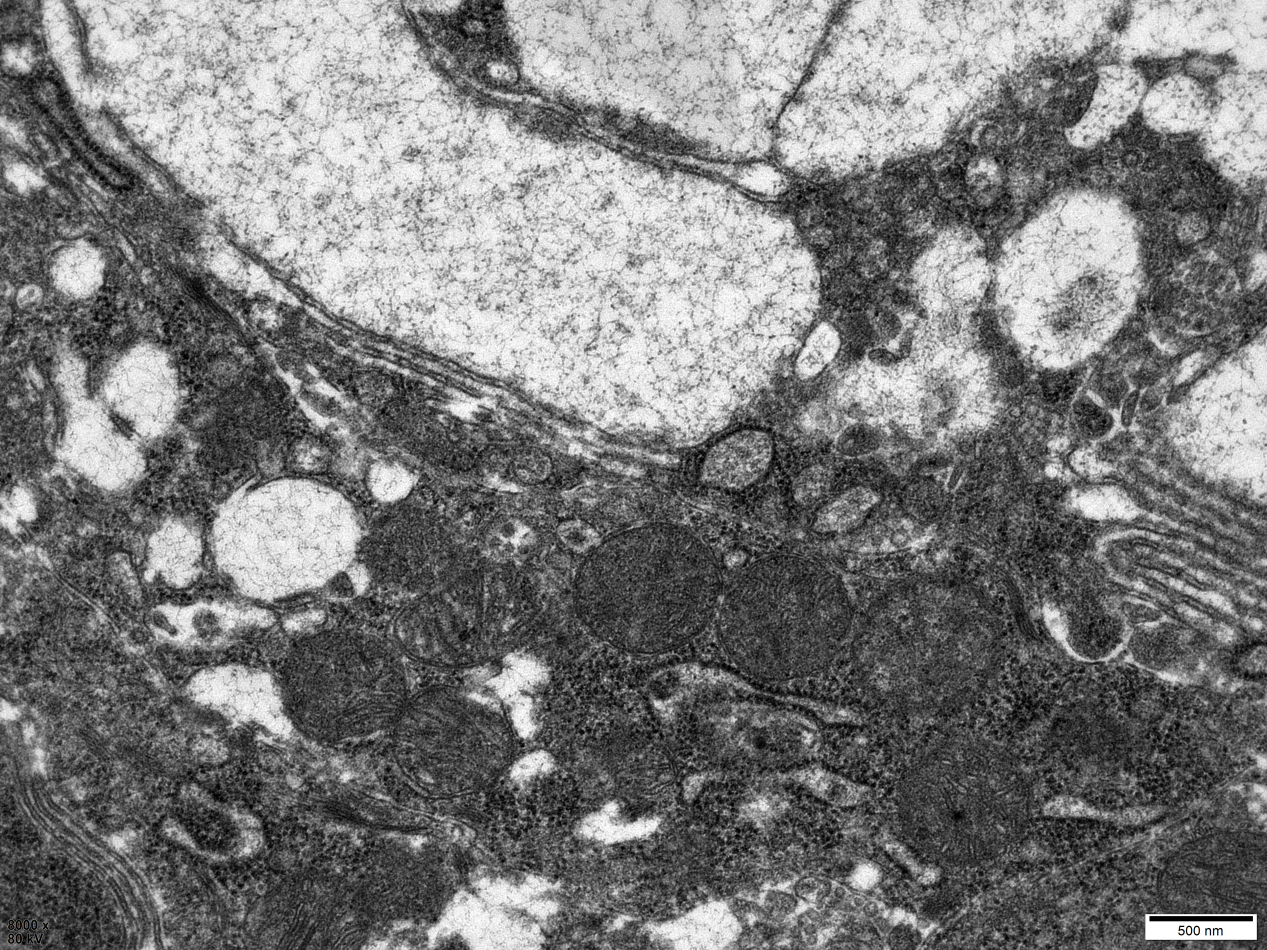


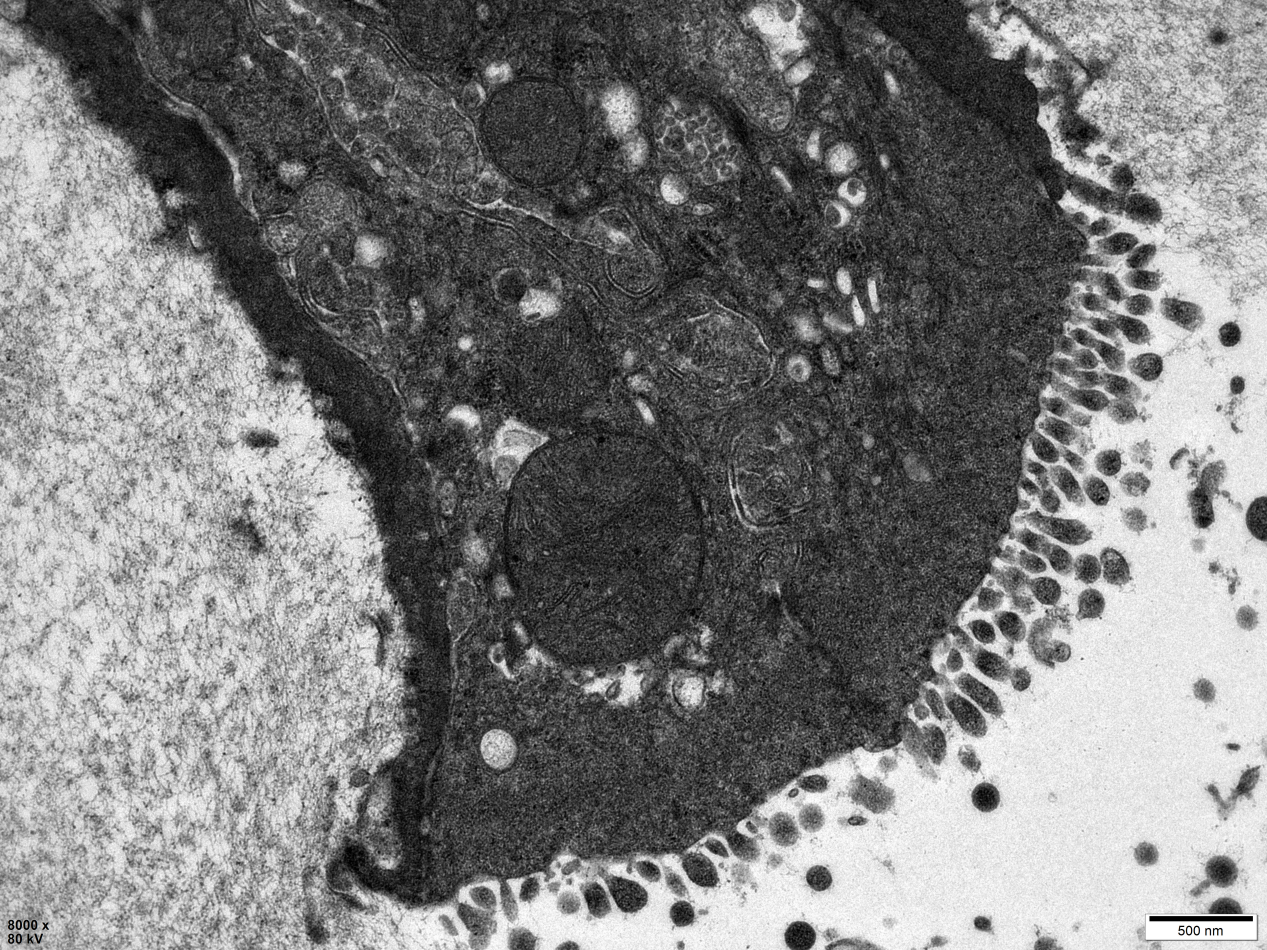


Model+Hirudin-Low


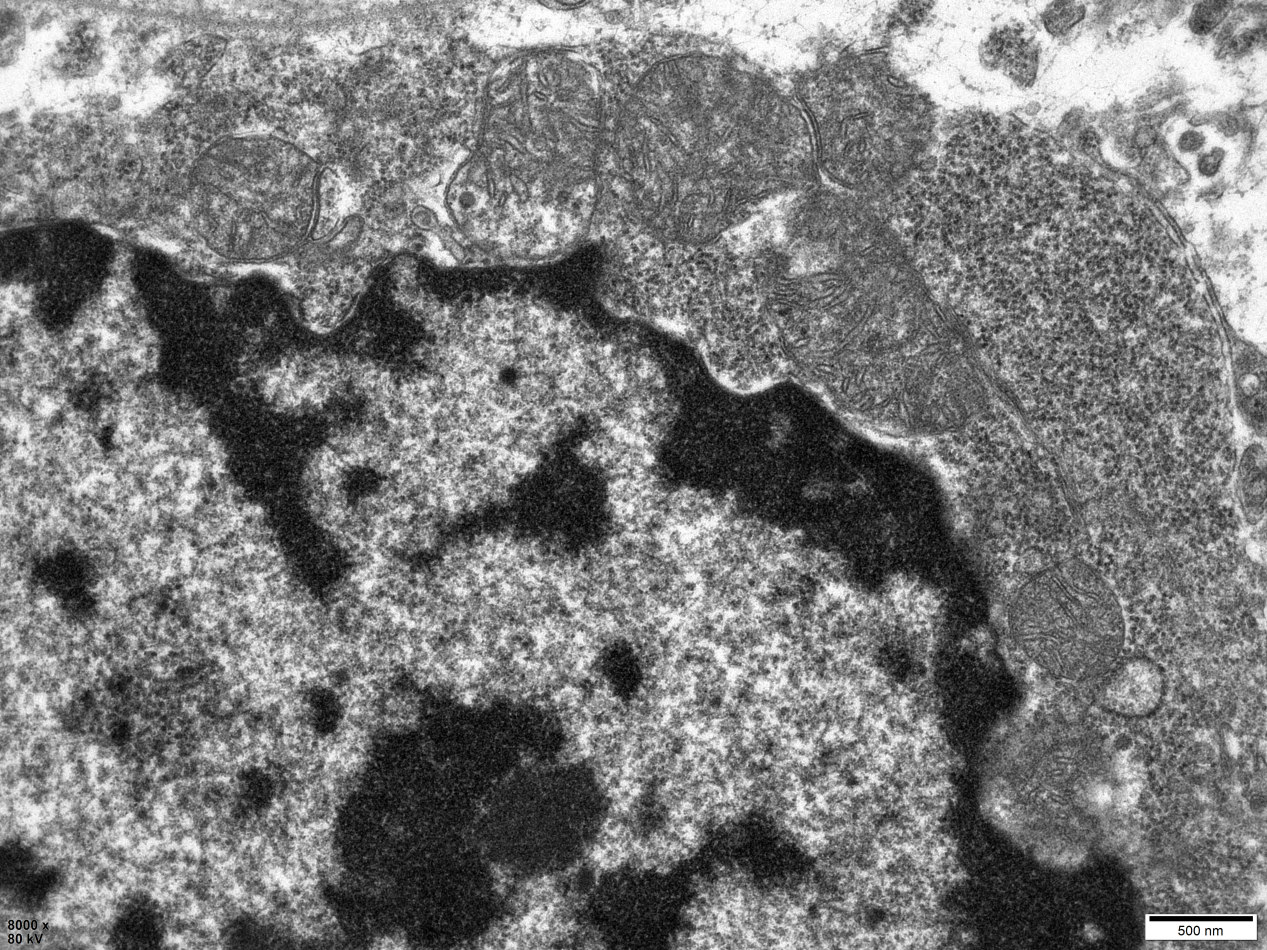


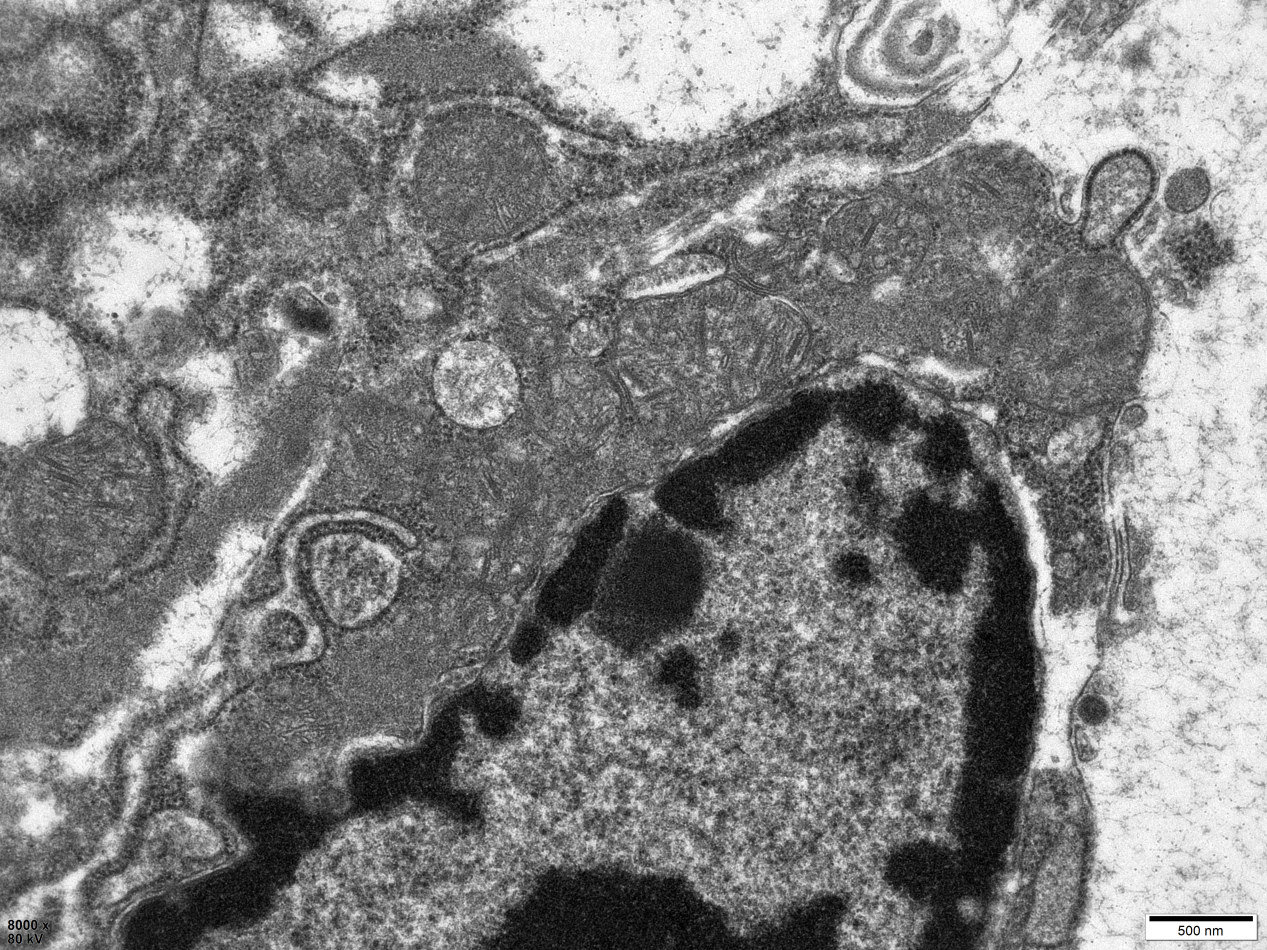


Model+Bifidobacterium


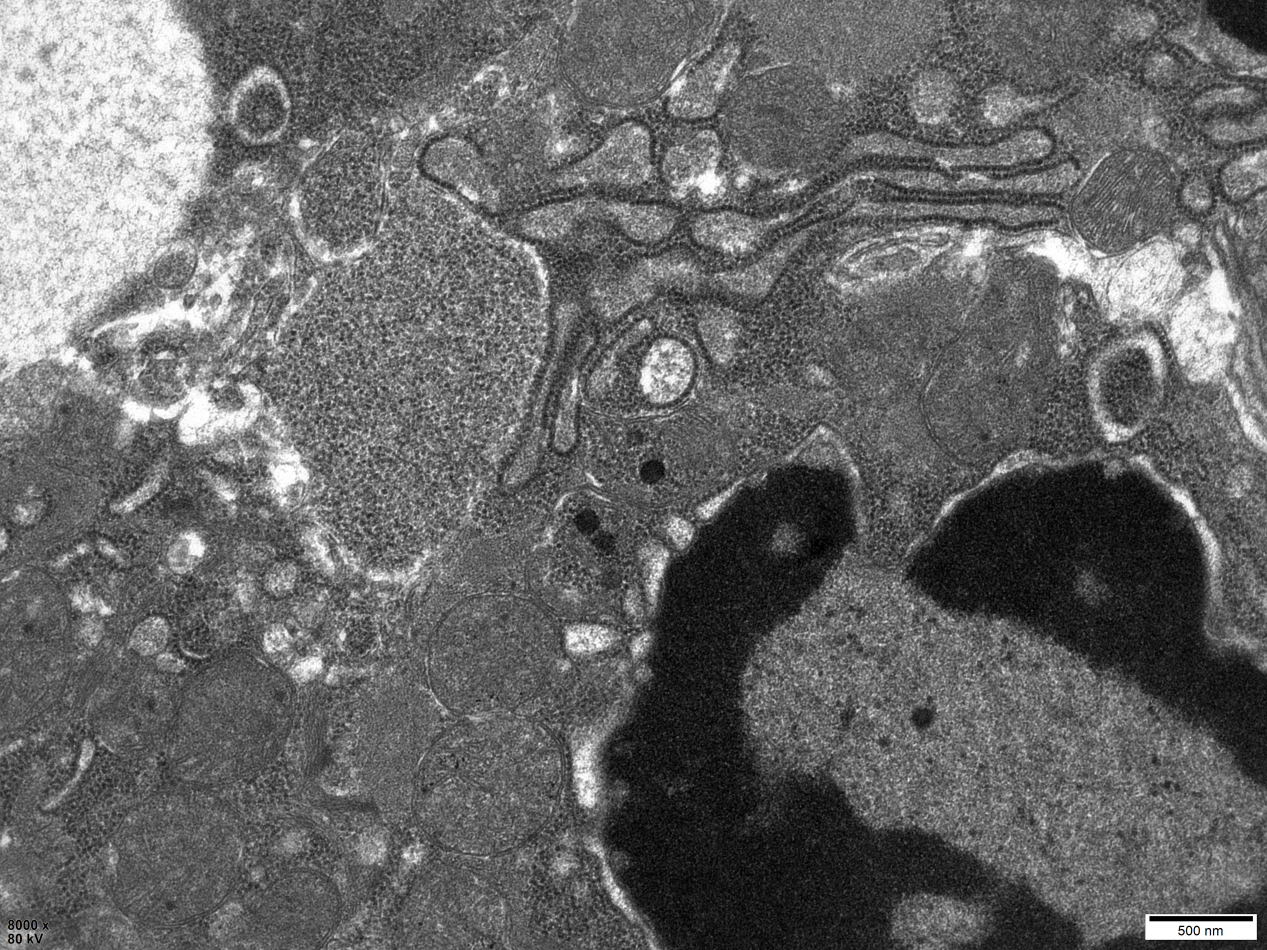


**
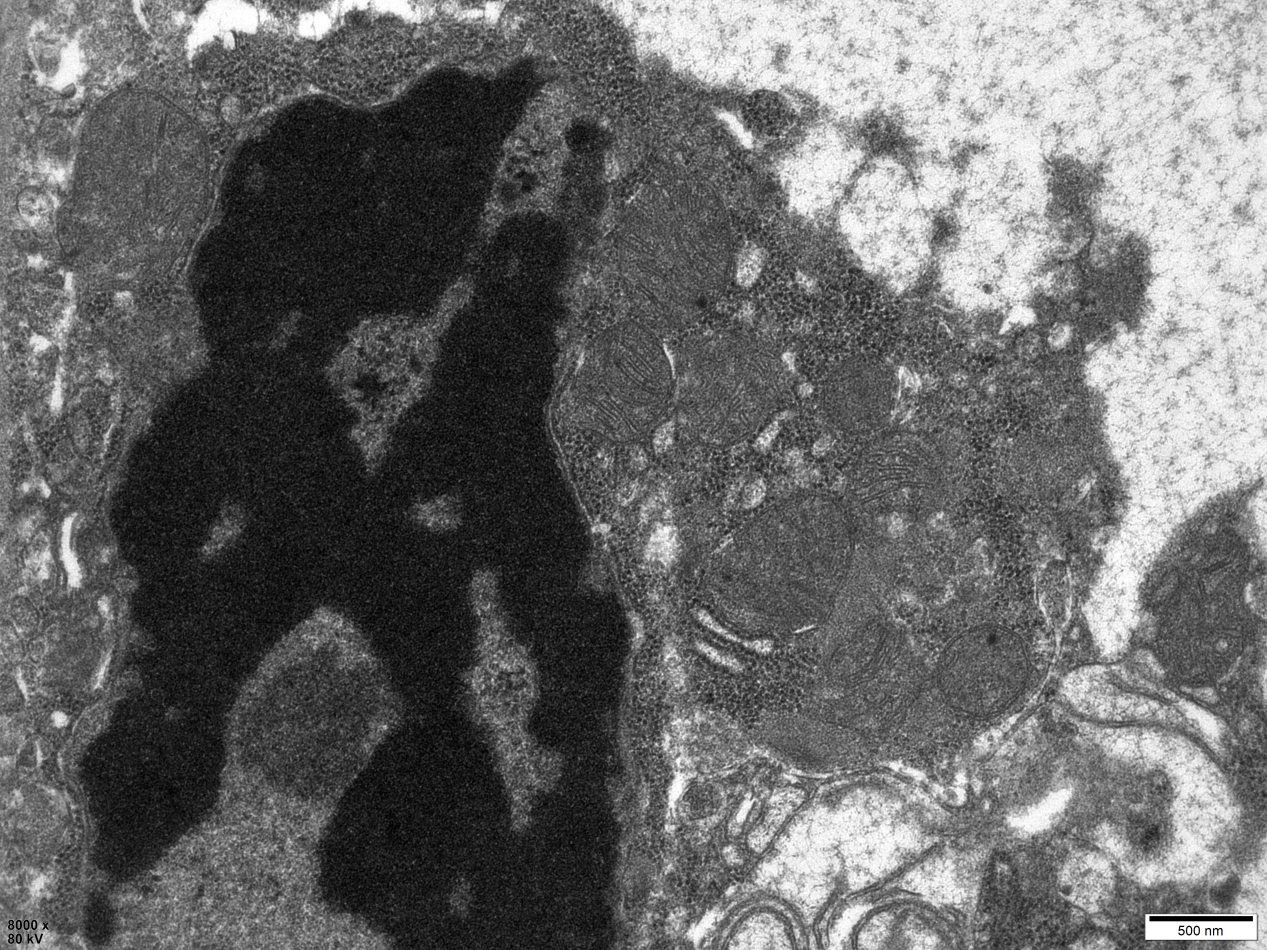
**

**Figure 5B**

NS


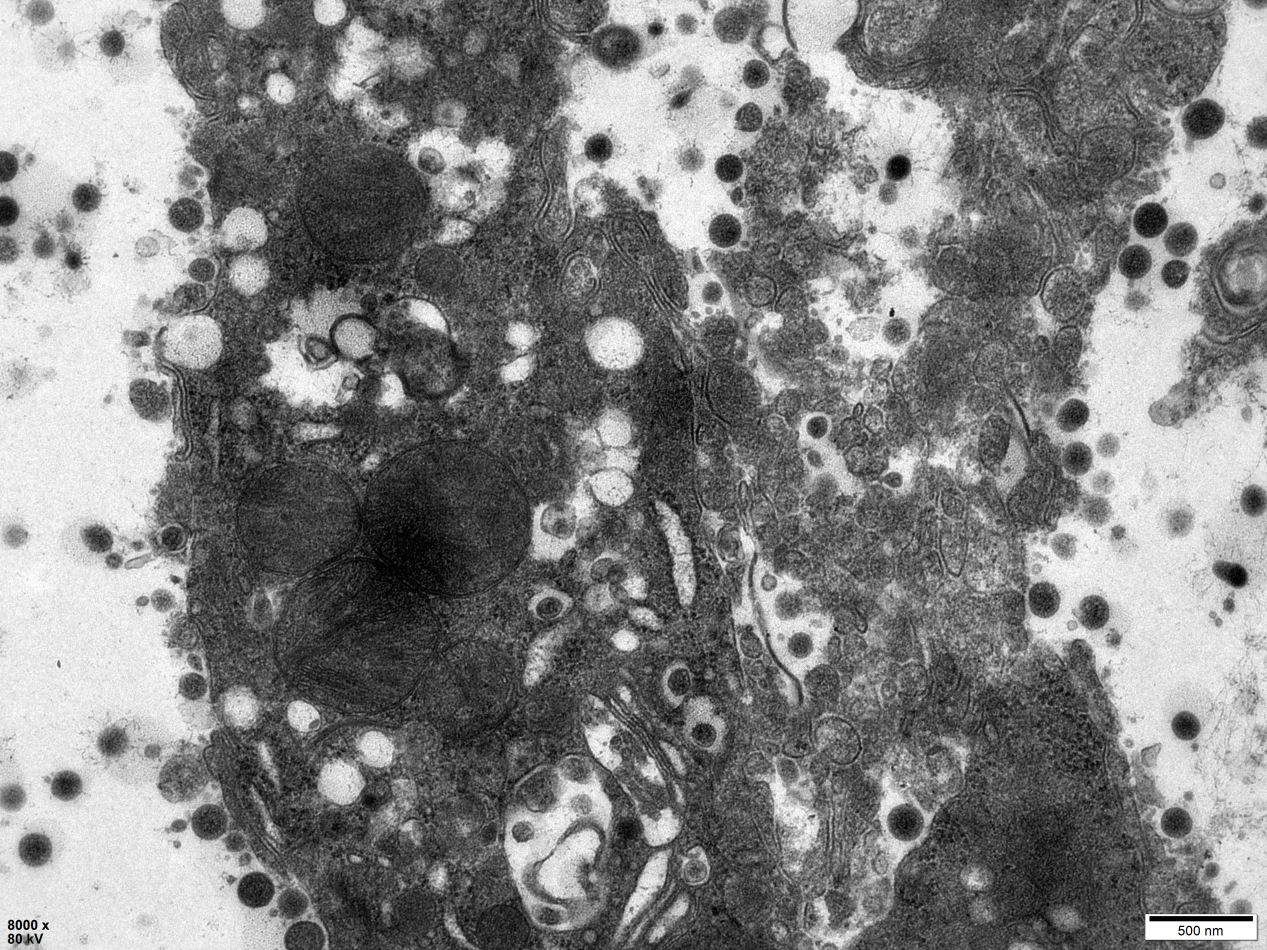


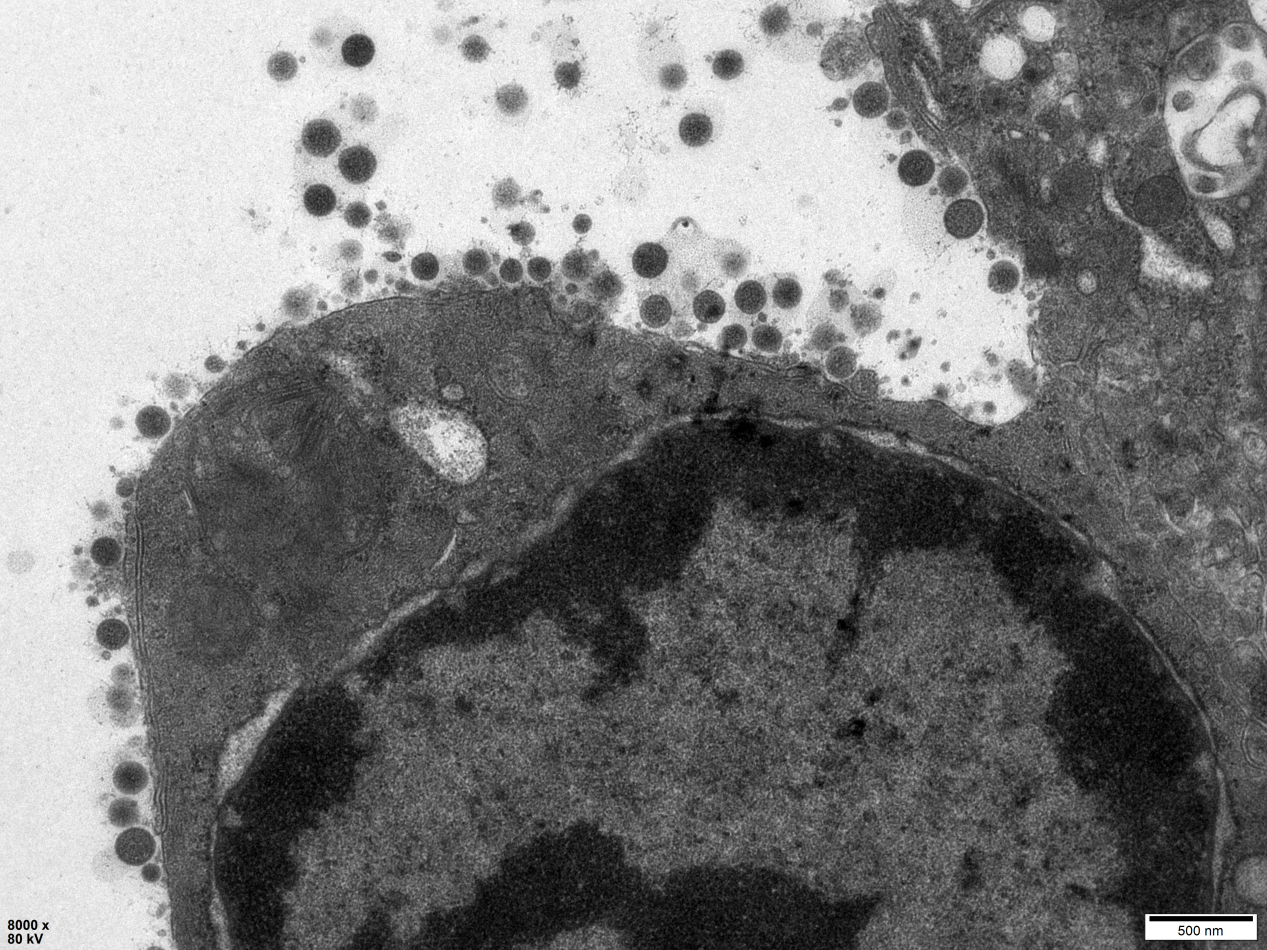


FMT


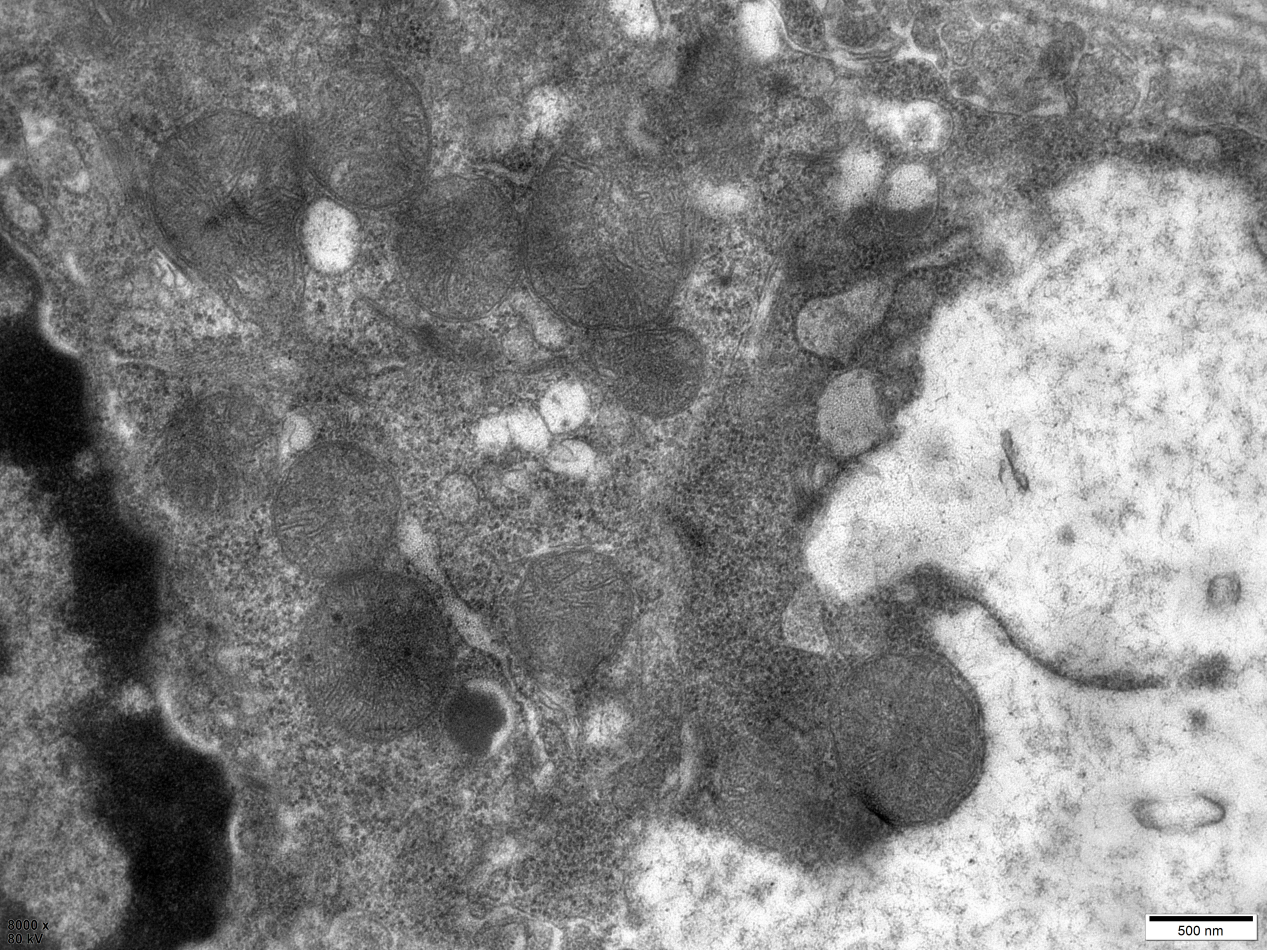


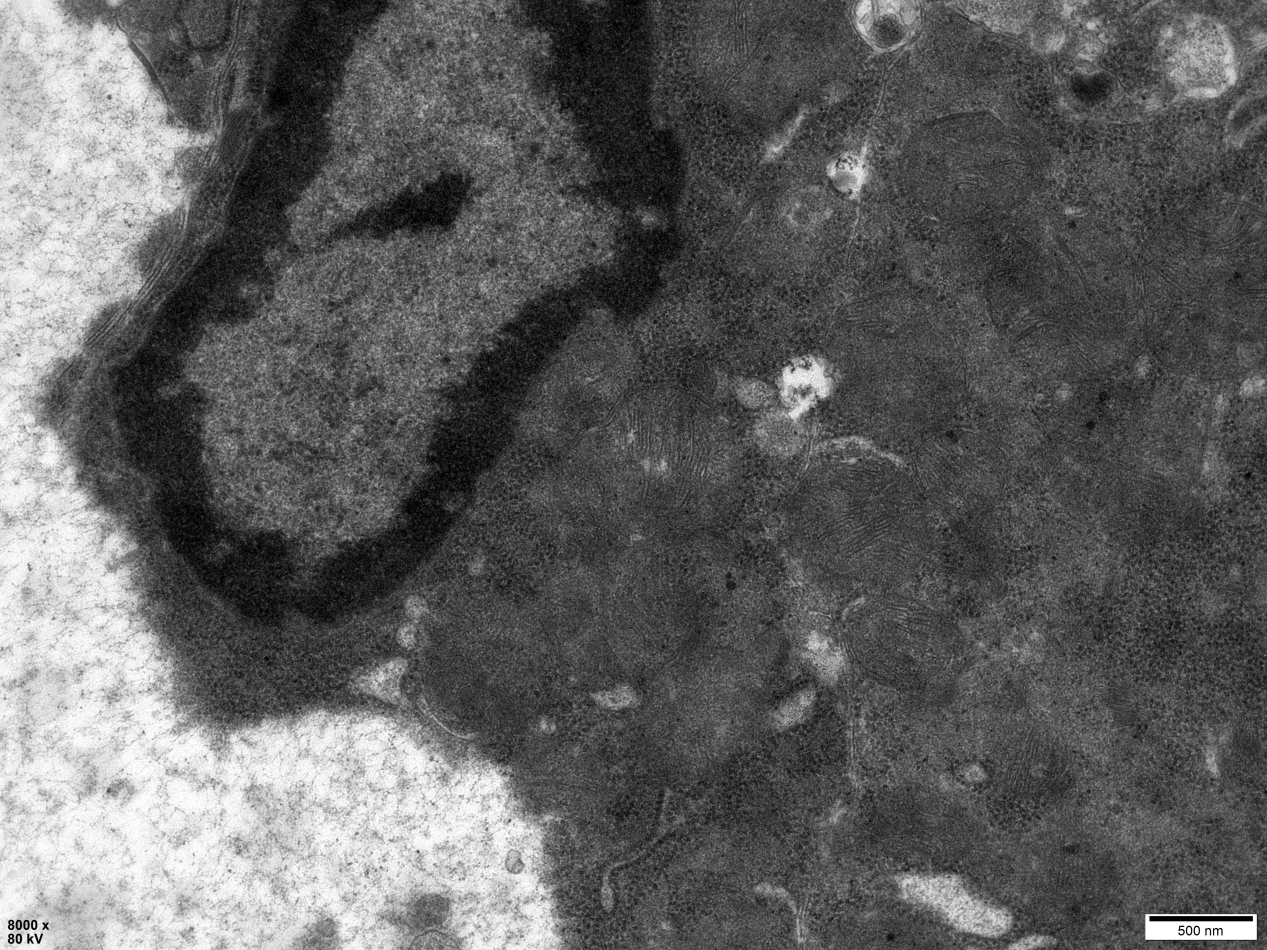

Supplement: Supplementary file 2 — Supplementary Material 2 [file 210_2023_2888_MOESM2_ESM.docx]
